# Adenosine-Triggered Dynamic and Transient Aptamer-Based Networks Integrated in Liposome Protocell Assemblies

**DOI:** 10.1021/jacs.5c05090

**Published:** 2025-05-22

**Authors:** Yu Ouyang, Yang Sung Sohn, Xinghua Chen, Rachel Nechushtai, Eli Pikarsky, Fan Xia, Fujian Huang, Itamar Willner

**Affiliations:** † Institute of Chemistry, The Hebrew University of Jerusalem, Jerusalem 91904, Israel; ‡ State Key Laboratory of Geomicrobiology and Environmental Changes, Faculty of Materials Science and Chemistry, 12564 China University of Geosciences, Wuhan 430074, China; § Institute of Life Science, The Hebrew University of Jerusalem, Jerusalem 91904, Israel; ∥ The Lautenberg Center for Immunology and Cancer Research, IMRIC, The Hebrew University of Jerusalem, Jerusalem 91120, Israel

## Abstract

The development of transient dissipative nucleic-acid–based reaction circuits and constitutional dynamic networks attracts growing interest as a means of emulating native dynamic reaction circuits. Recent efforts applying enzymes, DNAzymes, or light as catalysts controlling the transient, dissipative functions of DNA networks and circuits were reported. Moreover, the integration of the dynamic networks in protocell assemblies and the identification of potential applications are challenging objectives. Here, we introduce the adenosine (AD) aptamer subunit complex coupled with adenosine deaminase (ADA) as a versatile recognition/catalytic framework for driving transient allosterically AD-stabilized DNAzyme circuits or dissipative AD-stabilized constitutional dynamic networks. In addition, the AD/ADA-driven transient frameworks are integrated into liposome assemblies as protocell models. Functionalized liposomes carrying allosterically ATP-stabilized DNAzymes cleaving EGR-1 mRNA are fused with MCF-7 breast cancer cells, demonstrating effective gene therapy and selective apoptosis of cancer cells.

## Graphical abstract

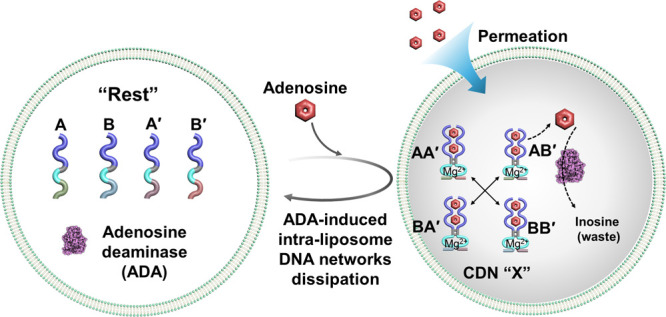

## Introduction

Native cellular transformations such as differentiation,^1^ migration,^2^ apoptosis,^3^ or metabolic pathways^4^ are guided and dictated by complex interconnected networks revealing signaling,^5^ sensing,^6^ adaptive,^7^ and cascaded^8^ features, exhibiting dynamic pathways including feedback,^9^ bistable,^10^ and oscillatory^11^ mechanisms controlled by equilibrated or out-of-equilibrium dissipative, transient frameworks. Indeed, substantial efforts are directed to develop synthetic networks emulating native systems as a part of the rapidly developing area of “System Chemistry”.^12^ Molecular,^13^ macromolecular,^14^ and protein assemblies^15^ mimicking basic functions of the native assemblies were reported. Particularly, the information encoded in the base sequences of nucleic acids, reflected by the precisely engineered of programmable reconfigurable oligonucleotide structures,^16^ and the signal-triggered switchable reconfiguration of nucleic acid structures by auxiliary stimuli, such as pH,^17^ metal ions,^18^ temperature,^19^ or light^20^ provide versatile tools to dynamically control synthetically engineered oligonucleotide biopolymers. Moreover, sequence-dictated recognition functions of oligonucleotides (aptamers)^21^ or catalytic properties of the biopolymer (DNAzymes)^22^ provide instructive means to fold or assemble, in the presence of auxiliary molecular/macromolecular ligands into supramolecular complexes or to catalyze programmed cleavage,^23^ nicking,^24^ or ligation^25^ of nucleic acids. In addition, sequence-dictated reactivities of oligonucleotides in the presence of enzymes, e.g., endonucleases,^26^ nickases,^27^ or polymerases^28^ allow the structural cut/paste/polymerization of the biopolymer. These versatile features of oligonucleotides provide a rich “tool-box” to assemble synthetic dynamic DNA machine^29^ and reconfigurable nanostructures^30^ and to identify potential sensing,^31^ biomedical,^32^ catalytic,^33^ and “smart” material applications. Furthermore, the structural and functional features of oligonucleotides were implemented to develop constitutional dynamic networks, CDNs,^34^ and transient, dissipative reaction circuits.^35^ Constitutional dynamic networks triggered by diverse auxiliary stimuli such as nucleic fuel strands,^36^ triplexes^37^ and light^38^ revealing adaptive,^36^ feedback,^39^ cascaded intercommunication,^37^ and orthogonal^40^ dynamic features were reported. Different applications of the CDNs were demonstrated, including CDN-guided biocatalysis,^41^ assembly of dynamic switchable soft materials for controlled drug release,^42^ and the use of CDN frameworks for biomedical therapeutic uses, e.g., dynamic switchable blood clotting^43^ or gene therapy.^44^ In addition, transient DNA-based circuits driven by enzymes,^45^ DNAzymes,^46^ light^47^ were engineered and exploited as functional circuits operating transient biocatalytic cascade^48^ and dissipative coagulation of fibrin.^40^ In addition, coupled, stimuli-responsive, switchable CDNs revealing transient behavior were demonstrated.^49^ The different dynamic networks and circuits were, however, operated in homogeneous aqueous phases, and their integration in confined, cell-like environments is desirable.

Substantial research efforts are directed to the development of microheterogeneous cell-like (protocell) assemblies.^50^ Liposomes,^51^ dendrosomes,^52^ polymersomes,^53^ proteinosomes,^54^ and phase-separated microdroplets^55^ were suggested as potential cell-like containments. Diverse catalytic and biocatalytic circuits were integrated in synthetic protocell assemblies, and the benefits of the confined reaction media on the respective chemical processes were highlighted.^56^ The integration of functional dynamic networks and transient operating circuits and their triggering by auxiliary external stimuli are still a challenge. The engineering of complex, multiconstituent networks in the confined environment, the effective signal-triggered activation of the networks, and the development of physical and spectroscopic means to follow and quantitatively probe the dynamic intraprotocell systems are still challenging barriers to overcome.

Here, we wish to report on the assembly of adenosine (AD)/AD-aptamer-subunit supramolecular complexes that allosterically stabilize functional DNAzyme structures and CDNs. The operation of the resulting catalytic DNAzymes or dynamic networks, in the presence of adenosine deaminase (ADA), leads to the concomitant transition of AD to inosine, resulting in the dynamic, transient, dissipative depletion of the AD-stabilized DNAzyme or AD-stabilized CDN. Moreover, a phototriggered pathway to evolve CDN is introduced. This involves the photochemical uncaging of two pre-engineered photoresponsive o-nitrobenzyl phosphate ester-caged hairpins that separate into four components equilibrating into a CDN upon photochemical deprotection. In addition, the AD-responsive DNAzyme reaction module and the AD-responsive CDN, in the presence of ADA, are integrated in liposome assemblies. Subjecting the loaded liposomes to AD as auxiliary stimuli results in the AD-triggered operation of the transient, dissipative AD-stabilized catalytic DNAzyme or the dynamically AD-controlled transient operation of the CDN in the liposome assemblies, acting as functional protocell model frameworks.

Moreover, the concepts introduced in the study demonstrating allosterically ligand/aptamer subunit-stabilized operation of circuits and networks in liposomes are extended to develop functional liposomes for gene therapy. In these systems, fusion of pre-engineered DNA nanostructure-loaded liposomes with cells leads to autonomous intracellular assembly of allosterically ATP-stabilized DNAzymes cleaving the EGR-1 mRNA, resulting in selective apoptosis of cancer cells.

## Results and Discussion

### Adenosine-Triggered Transient Dynamic DNA Circuits and Dynamic Constitutional Networks

In the first step, we confirmed the application of the adenosine (AD) ligand/aptamer subunit complex and coupled ADA as functional constituents driving a dissipative, transient, reaction circuit (Figure 1). The “Rest” reaction module includes nucleic acid constituents (L_1_/F and L_2_/Q) that the L_1_ and L_2_ comprise two antiadenosine aptamer units (a_1_, a_2_) and hybridize with the FAM fluorophore-modified strand (F) and the BHQ1 quencher-modified strand (Q), and the ADA that transform AD to inosine, respectively. Subjecting the “Rest” reaction module to AD results in the assembly of the supramolecular complex consisting of the AD/AD-aptamer subunits, in which the spatial proximity between the fluorophore and quencher associated with L_1_ and L_2_ leads to quenching of the fluorescence of F. The simultaneous ADA-catalyzed conversion of AD to inosine results in the disassembly of the supramolecular intermediate, as inosine lacks affinity for the aptamer subunits. This process results in the transient restoration of the parent reaction module and the recovery of the fluorescence characteristic of the “Rest” module. That is, triggering the “Rest” reaction module with AD yields the temporal assembly of the intermediate AD/(L_1_F+L_2_Q) complex and concomitant ADA-stimulated transient recovery of the “Rest” reaction circuit. The dynamic dissipative process is, then, followed by the transient fluorescence changes. Figure 1B, Panel I depicts the transient fluorescence intensities of the system upon subjecting the “Rest” reaction circuits to variable concentrations of AD in the presence of a constant concentration of ADA (0.025 U/mL). As the concentration of AD increases, the recovery time intervals of the “Rest” module are prolonged. Figure 1B, Panel II presents the transient fluorescence intensities of the system using a fixed concentration of AD (4 mM) to trigger the process in the presence of variable concentrations of ADA. As the concentration of ADA increases, the transient recovery of the “Rest” reaction circuit is faster. By applying a calibration curve relating the fluorescence intensities to variable concentrations of the AD/(L_1_F+L_2_Q) complex, Figure S1 and accompanying discussion, the transient fluorescence intensities of the system shown in Figure 1B were translated to transient concentration changes of the intermediate AD/(L_1_F + L_2_Q) complex, generated upon triggering the reaction module with variable concentrations of AD (fixed ADA concentration, 0.025 U/mL), Figure 1C, Panel I, and the transient concentration changes of AD/(L_1_F + L_2_Q) upon subjecting the reaction module to a fixed concentration of AD (4 mM), and variable ADA concentrations, Figure 1C, Panel II. It should be noted that the AD aptamer reveals affinity to other adenine-containing ligands, such as AMP, ADP, or ATP. Nonetheless, foreign nucleotides, including CTP, TTP, GTP, and UTP, lack any affinity toward the AD aptamer subunits (see Figure S2 and accompanying discussion). Moreover, the results depicted in Figure 1 demonstrated that the reaction module consisting of duplexes L_1_/F, L_2_/Q, and ADA is triggered by AD to yield a supramolecular transient complex that is transduced by its fluorescent features. The transient behavior of the complex was controlled by the concentration of ADA, thus suggesting that the system could act as an ADA sensing platform. In fact, the quantitative detection of ADA has clinical diagnostic significance. Elevated amounts of ADA were found in the serum of esophagus cancer, gastric cancer, and pancreatic cancer carriers,^57^ and ADA deficiency was found to cause severe combined immunodeficiency.^58^ Indeed, several analytical methods, including high-performance liquid chromatography,^59^ a colorimetric gold nanoparticle aptamer assay,^60^ and a label-free fluorescent DNA-templated silver nanocluster platform,^61^ were reported to detect ADA. Figure S3 and the accompanying discussion present the application of the reaction module presented in Figure 1 to sense ADA. The sensing platform allows the detection of ADA with a sensitivity corresponding to 0.0125 U/mL, a value superior or comparable to the reported methods.

**Figure 1:**
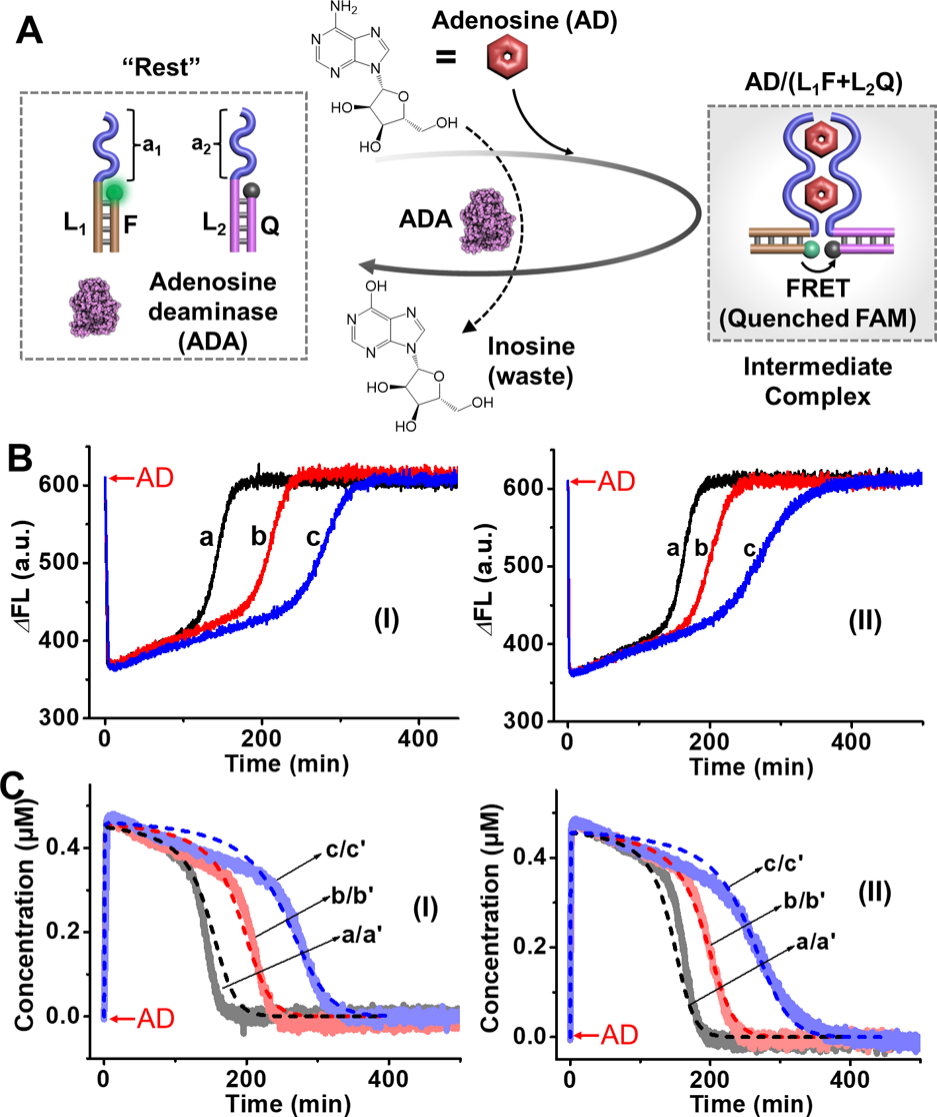
(A) Schematic assembly of a dissipative adenosine (AD)/adenosine deaminase (ADA) reaction circuit assembling a supramolecular AD/AD-aptamer complex as an intermediate. The transient operation of the system is followed via the FRET signal transduced by the complex. (B) Transient fluorescence changes of the AD/AD-aptamer subunits supramolecular complex upon operating the circuits shown in (A). Panel I-in the presence of ADA, 0.025 U/mL, and variable concentrations of AD: (a) 3 mM, (b) 4 mM, (c) 5 mM; Panel II-in the presence of AD, 4 mM, and variable concentrations of ADA: (a) 0.033 U/mL, (b) 0.025 U/mL, (c) 0.017 U/mL. (C) Transient concentration changes of the AD/AD-aptamer subunits supramolecular complex upon operating the circuits shown in (A). Panel I-in the presence of ADA, 0.025 U/mL, and variable concentrations of AD: (a) 3 mM, (b) 4 mM, (c) 5 mM; Panel II-in the presence of AD, 4 mM, and variable concentrations of ADA: (a) 0.033 U/mL, (b) 0.025 U/mL, (c) 0.017 U/mL.

Moreover, throughout the study, including the system depicted in Figure 1, AD-triggered AD-aptamer subunit complexes guide, in the presence of ADA, the different transient reaction circuits. In these processes, ADA catalyzes the deamination of AD to inosine, resulting in the separation of the AD from the AD-aptamer subunit complexes. It is, however, important to evaluate possible binding and associated interferences to the systems through binding of inosine or ADA to the aptamer subunits. Figure S4 shows the binding properties of AD to the aptamer subunits in the presence of ADA and inosine. Evidently, neither ADA nor inosine has any perturbations toward binding of AD to the aptamer subunits (*K*_d_ = 11.8 ± 2.3 μM). In addition, inosine and ADA by themselves lack any affinity for the aptamer subunits, indicating the stability of the AD/aptamer subunits complex and the absence of inosine-induced interference in the complex formation. This stability allows the recycling of transient AD-driven processes, as shown in Figure S5.

The experimental transient concentration changes of the intermediate product shown in Figure 1C were computationally simulated. To achieve this goal, a kinetic model for the circuit shown in Figure 1A was developed, incorporating the various rate constants associated with the reaction scheme depicted in Figure S6. Taking into account the experimental *K*_d_ value, corresponding to the dissociation constant of AD to the aptamer subunits (*K*_d_ = *k*_1_/*k*_–1_, 11.8 μM) in the model, curve (b) shown in Figure 1C, Panel I, was computationally simulated to obtain the best fit overlay curves, curves (b′). The rate constants for the best fit overlaid curve are summarized in Table S1. The set of rate constants was then employed to predict the dynamic behavior of the system at different auxiliary conditions (different concentrations of AD and ADA) displayed in curves a′ and c′ (dashed lines) in Figure 1C, Panels I and II. The predicted transient dynamic behavior of the intermediate product was then experimentally validated, curves b and c. The resulting very good fits between the predicted and experimentally validated results suggest that the kinetic model is able to describe the reaction module and emphasize the significance of computational simulations to predict the behaviors of the dynamic circuit at different conditions. This procedure was adopted to construct kinetic models for the additional dynamic systems discussed in the study, which will be addressed in subsequent sections. (cf. Figures 2–4).

**Figure 2:**
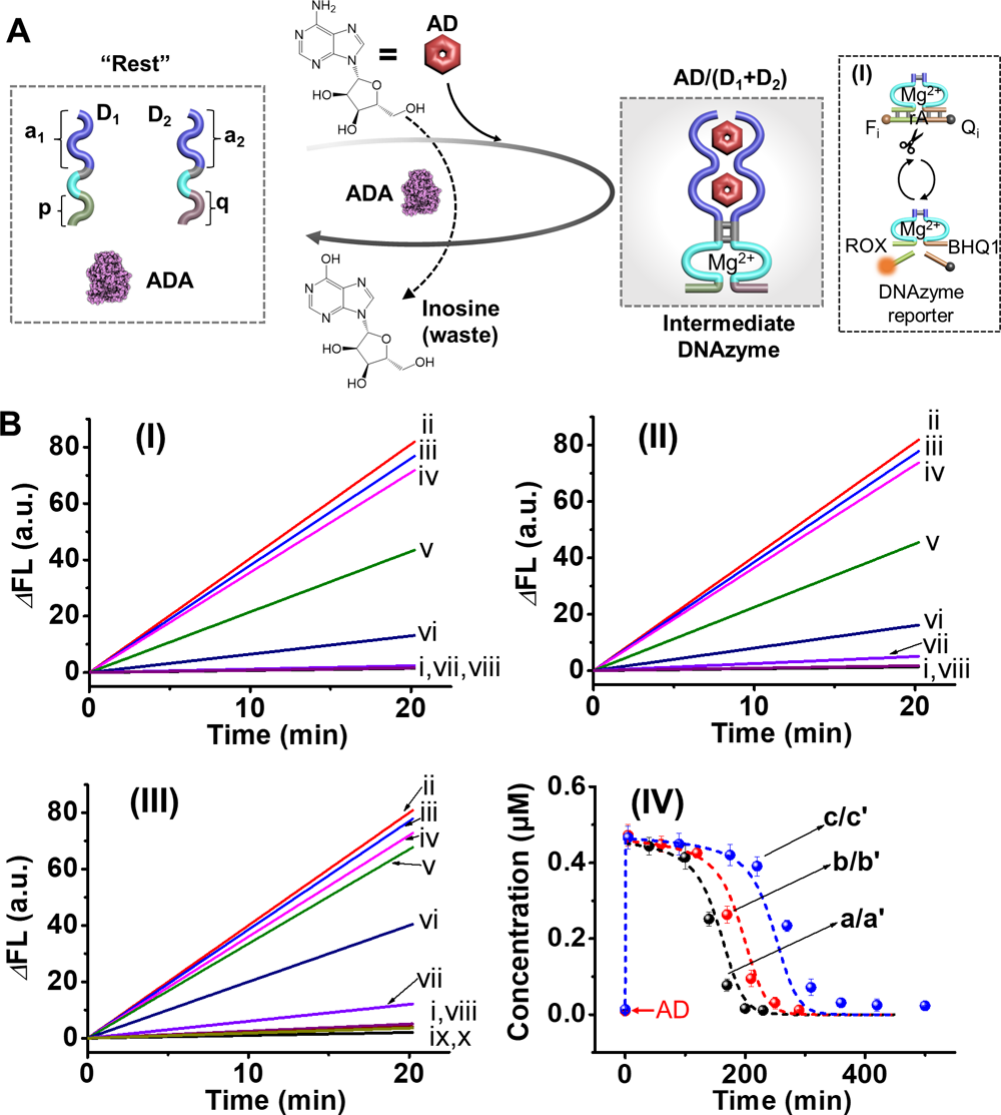
(A) Schematic reaction module presenting the AD/ADA-driven transient formation and depletion of an AD/AD-aptamer subunit-stabilized Mg^2+^-ion-dependent DNAzyme. Inset: the transient catalytic activities of the DNAzyme are probed by the DNAzyme-catalyzed cleavage of the F_i_ (FAM)/Q_i_ (BHQ1)-functionalized ribonucleobase-modified substrate. (B) Time-dependent fluorescence intensity changes generated by the DNAzyme-catalyzed cleavage of the F_i_/Q_i_-modified substrate by 30 μL samples withdrawn from the reaction mixture during operation of the transient catalytic circuit at time intervals: Panel I-(i) 0, (ii) 5, (iii) 40, (iv) 100, (v) 140, (vi) 170, (vii) 200, and (viii) 230 min.; Panel II-(i) 0, (ii) 5, (iii) 60, (iv) 120, (v) 170, (vi) 210, (vii) 250, and (viii) 290 min; Panel III-(i) 0, (ii) 5, (iii) 90, (iv) 175, (v) 220, (vi) 270, (vii) 310, (viii) 360, (ix) 420, and (x) 500 min. Panel I, reaction circuit activated by AD, 3 mM. Panel II, reaction circuit activated by AD, 4 mM. Panel III, reaction circuit activated by AD, 5 mM. Panel IV, transient concentration changes of the AD-stabilized Mg^2+^-ion-DNAzyme in the presence of (a) AD, 3 mM; (b) AD, 4 mM; (c) AD, 5 mM. (Solid dots correspond to the experimental concentration changes of the AD-stabilized DNAzyme transduced from the rates of cleavage of the DNAzyme substrate within the samples withdrawn from the reaction solution shown in Panels I–III; dash curves-simulation results.) Error bars are derived from three independent experiments.

**Figure 3:**
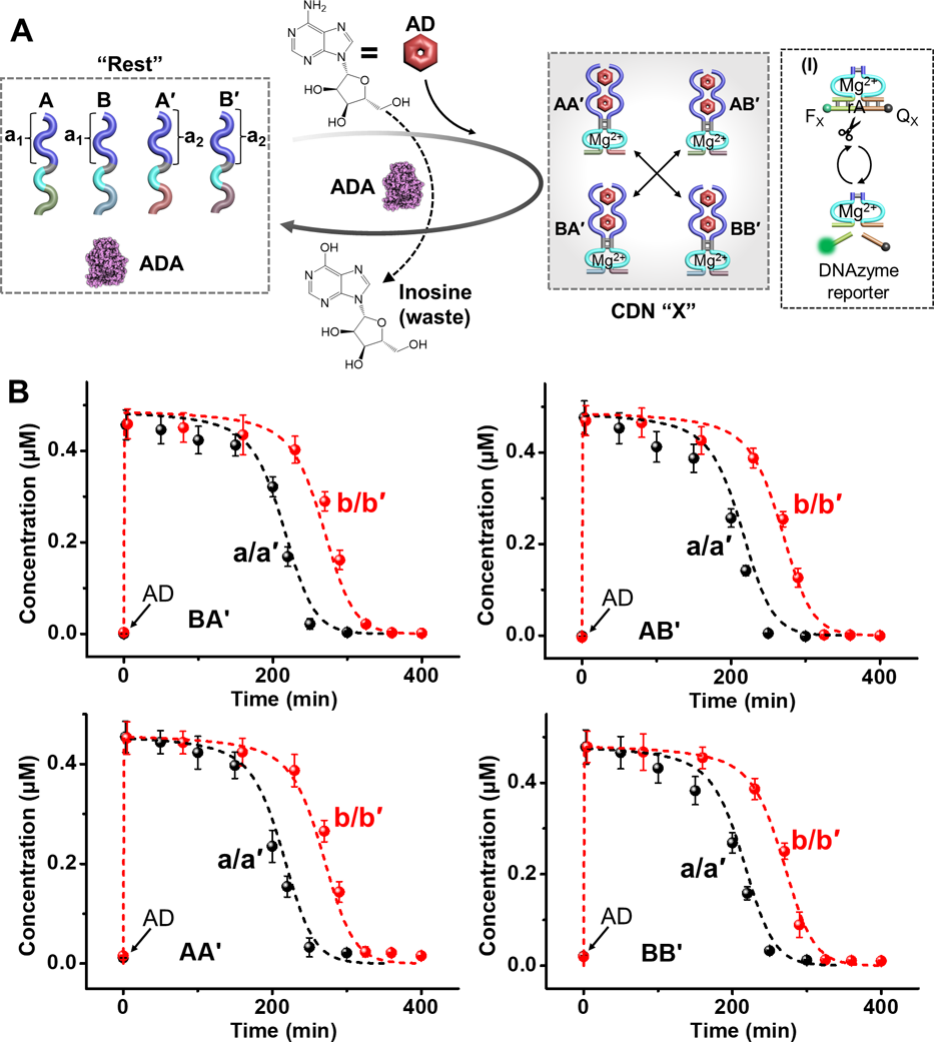
(A) Schematic assembly of a reaction module consisting of four nucleic acid components triggered by AD to yield a transiently operating AD-stabilized constitutional dynamic network. The dynamic operation of the network is probed by Mg^2+^-ion-dependent DNAzyme reporter units conjugated to the temporally generated constituents, Panel I. (B) Transient time-dependent concentration changes of the constituents AA′, AB′, BA′, and BB′ in reaction samples withdrawn at time intervals from the dissipative operating network shown in (A) in the presence of different concentrations of AD (a/a′ AD = 4 mM, b/b′ AD = 5 mM). Solid points in (a) and (b) correspond to experimental data; dashed lines in (a) and (b) correspond to computationally simulated data. Error bars are derived from three independent experiments.

**Figure 4:**
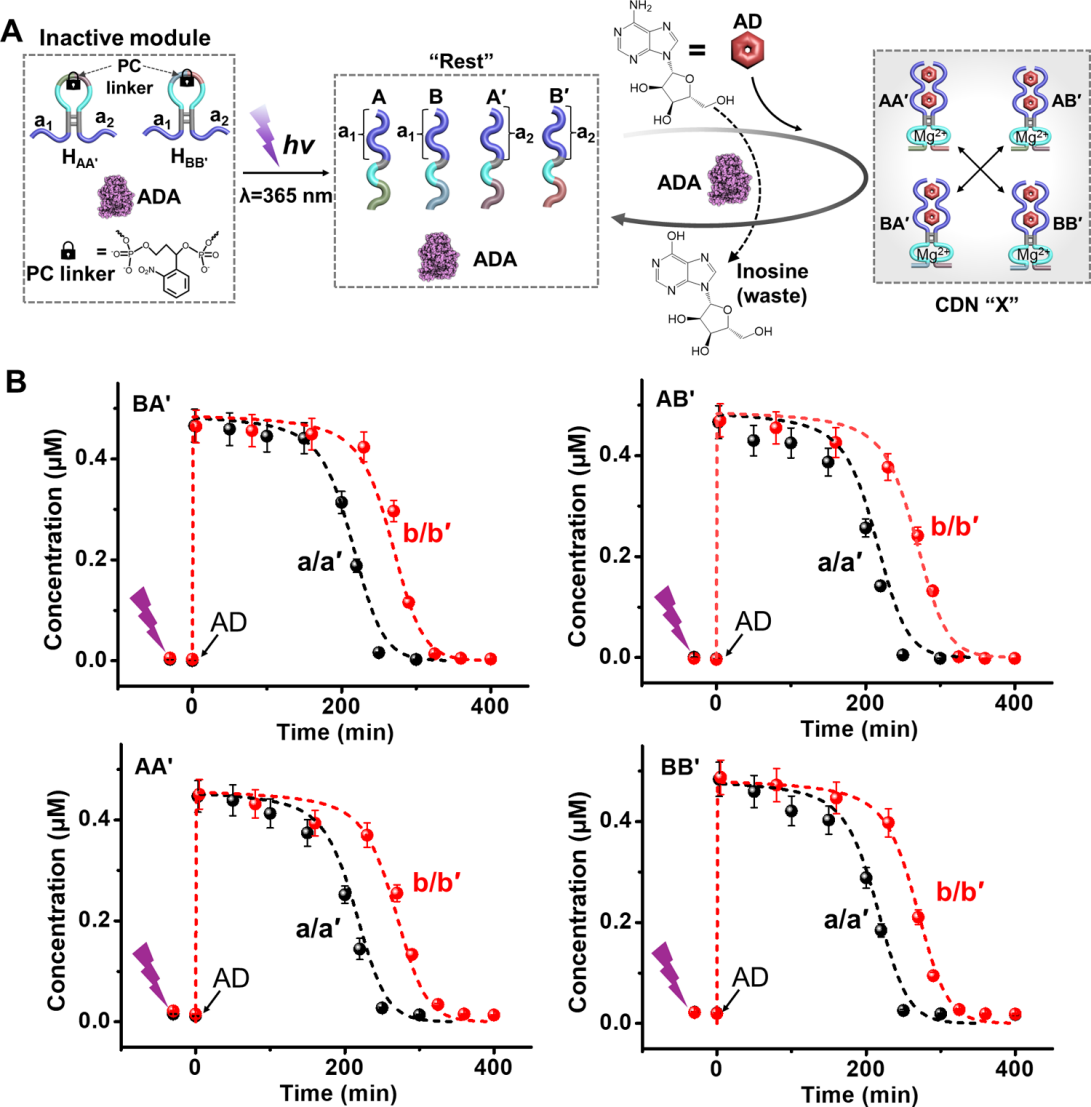
(A) Schematic reaction module for the photochemically triggered evolution of AD-fueled stabilization of a transiently operating constitutional dynamic network (CDN X). The transient operation of the dynamic network is probed by Mg^2+^-ion-dependent DNAzyme reporter units conjugated to the temporally generated constituents of CDN X. (B) Transient concentration changes of the constituents comprising CDN X in samples withdrawn at time intervals from the reaction mixture shown in part A, in the presence of ADA, 0.025 U/mL, and different concentrations of AD: a/a′, 4 mM; b/b′, 5 mM. Solid points in part a correspond to experimental data; dashed lines in part a correspond to computationally simulated data. Error bars are derived from three independent experiments.

The assembly of an AD/AD-aptamer-subunits complex and its separation by ADA yielding a dissipative structure was applied to design a functional transient DNAzyme catalyst, where the AD/AD-aptamer-subunits complex allosterically guides the transient catalytic functions of a Mg^2+^-ion-dependent DNAzyme, Figure 2A. The “Rest” reaction module consists of auxiliary ADA, and two DNA strands D_1_ and D_2_ that include the aptamer subunits (a_1_ and a_2_) conjugated to tethers (p, q) comprising the Mg^2+^-ion-dependent DNAzyme subunits. Subjecting the reaction module to AD results in the assembly of active DNAzyme that cleaves the fluorophore (ROX-F_i_)/quencher (BHQ1-Q_i_)-modified substrate, Panel I. The concomitant ADA-catalyzed transformation of AD to inosine separated the DNAzyme structure while restoring the mute “Rest” system. By sampling the catalytic activities of the DNAzyme at time intervals during operation of the reaction circuit, the transient catalytic features of the intermediate DNAzyme are probed. Figure 2B, Panels I–III, depict the rates of cleavage of the F_i_/Q_i_-substrate by the temporally generated DNAzyme in samples withdrawn from the reaction mixture in the presence of different AD fuel concentrations. Using an appropriate calibration curve relating the catalytic rate of substrate cleavage to the concentration of the DNAzyme, Figure S7, the dynamic transient concentrations of the DNAzyme in the presence of different AD concentrations were evaluated, and these are displayed in Figure 2B, Panel IV. As the concentration of AD increases, the lifetime of the transient intermediate DNAzyme is prolonged. The experimental transient evolution and depletion of the AD-stabilized Mg^2+^-ion-dependent DNAzyme were computationally simulated (see the formatted kinetic model and derived rate constants for the reaction circuits, Figure S8 and Table S2). The computationally fitted transient curve b′ (dotted line) is overlaid on the experimental data, curve b (solid points). The derived rate constants were employed to predict the transient behavior of the circuits in the presence of different concentrations of AD, curves a′, c′ (dotted line), and the computational results were validated experimentally, as shown in curves a and c (solid points). AD-guided transient DNAzyme circuitry reveals functional sustainability. By readdition of the AD ligand, the transient circuitry can be recycled with no noticeable loss of its catalytic function, Figure S9 and accompanying discussion. Furthermore, the allosterically AD-induced activation of the Mg^2+^-ion-dependent DNAzyme and the demonstrated affinity of the AD-aptamer subunits toward ATP, cf. Figure S2 paved the way to engineer an allosterically ATP-driven DNAzyme for intracellular cleavage of the EGR-1 mRNA and resulting selective apoptosis of MCF-7 breast cancer cells, vide infra and see Figures S22 and S23 and accompanying discussion.

The ability to induce by AD and aptamer-subunits a supramolecular AD/aptamer subunit complex and induce the transient separation of the complex in the presence of ADA was further applied to develop an AD-driven dynamic assembly and transient disassembly of a transient operating constitutional dynamic network (CDN), Figure 3A. Four nucleic acid strands A, A′, B, B′ that include each the AD binding subunit (a_1_, a_2_) are extended by pre-engineered nucleic tethers that include Mg^2+^-ion-dependent DNAzyme subunits. In addition, the “Rest” reaction circuit includes ADA as an internal biocatalyst. Subjecting the reaction circuit to AD results in the assembly of four equilibrated constituents, AA′, AB′, BA′, and BB′, stabilized each by the AD/aptamer-subunit complex, while stabilization of the AD/aptamer subunits allosterically stabilizes a Mg^2+^-ion-dependent DNAzyme within the constituent structures. The ADA integrated in the system catalyzes the transformation of AD to inosine, resulting in the separation of the constituents and the transient recovery of the parent “Rest” systems. The Mg^2+^-ion-dependent DNAzyme associated with the constituents includes different binding “arms” of the substrate for cleavage of the respective fluorophore (F_*x*_)/quencher (Q_*x*_)-modified ribonucleobase substrates, Panel I. The DNAzyme units act as quantitative “reporter” units that probe quantitative contents of the constituents in the CDN X by following the rates of cleavage of the DNAzyme substrates and using appropriate calibration curves relating the catalytic rates to the concentration of the respective DNAzymes. Accordingly, the AD-triggered formation of the intermediate AD-stabilized CDN X and the dynamic, transient, ADA-stimulated recovery of the “Rest” reaction module are quantitatively evaluated by the DNAzyme “reporter” units, associated with the constituents, probing at time intervals the temporal concentrations of the constituents in the dynamic CDN X system. Figure S10 presents the rates of cleavage of the F_*x*_/Q_*x*_-modified substrates by the respective DNAzyme “reporter” units in samples withdrawn at time intervals from the transient dynamic evolution of the CDN X reaction triggered with different concentrations of AD. Figure S11 shows the relation between the concentrations of “intact” DNAzyme “reporter” units and the cleavage rates of their substrates and presents the calibration curves illustrating the relationship between the catalytic rates of substrate cleavage and the concentrations of the DNAzymes. Using these calibration curves, the temporal, transient concentrations of the constituents upon the AD-triggered assembly of CDN X and its dynamic concomitant ADA-induced depletion, in the presence of two concentrations of AD, were evaluated. Namely, aliquots of samples were withdrawn at time intervals of operating CDN X in the presence of two different concentrations of AD to evaluate the temporal concentrations of the constituents at different time intervals of transient operation (the times for withdrawing the samples from the reaction mixtures were guided by the temporal behavior of the circuitry shown in Figure 1C to follow the overall transient behaviors of the systems). The results are displayed in Figure 3B demonstrating the AD/ADA-coupled transient concentrations of the constituents AA′, AB′, BA′, and BB′ in the temporally operating CDN X. As the concentration of AD is higher, the depletion of the constituents of CDN is enhanced. (For the effects of auxiliary parameters, such as Mg^2+^-ion concentrations or temperature, on the function of CDN X, see Figures S12 and S13. Also, for the stability of CDN X and its function, see Figure S14 and the accompanying discussion.) The experimental transient evolution and depletion of the AD-stabilized CDN X were computationally simulated. (See the formatted kinetic model and derived rate constants for transient dynamic networks, Figure S15 and Table S3). The derived rate constants were employed to predict the transient behavior of the transient operation of CDN X in the presence of additional concentrations of AD, curves b′, and the computational results were validated experimentally, as shown in curves b (solid points).

The different systems discussed throughout this paper made use of the AD trigger and the specific AD-aptamer interaction as a powerful method to guide the transient operation of constitutional dynamic networks and of transient circuits. While the selectivity introduced by the AD-ligand interaction is certainly an advantage of the systems, it raises the question whether auxiliary added components could inhibit and eventually modulate the dynamic circuits and networks. In fact, a versatile means to develop potential inhibitors toward the specific AD-aptamer interaction could involve the use of DNA strands complementary to the aptamer domains that perturb the binding of the AD trigger activating the circuits. This is exemplified with the inhibition of the AD-triggered transient formation of the supramolecular complex AD/(L_1_F + L_2_Q), cf. Figure 1A, see Figure S16 and accompanying discussion, and of the CND X, cf. Figure 3A, see Figure S17 and accompanying discussion, upon addition of an auxiliary strand that hybridizes to the aptamer binding domain.

Moreover, an end goal of the study is to integrate the AD/ADA-triggered, transient operation of the circuits in “protocell” liposome assemblies (vide infra) and particularly the dynamic transient operation of a CDN in liposomes. This forced us to design a method for assembling a “simple″ caged reaction circuit that enables the triggered formation of a precisely controlled concentration-dependent assembly of the four strands shown in Figure 3A, within a confined reaction medium. Toward this goal, we designed two photoresponsive o-nitrobenzyl phosphate ester-caged^62^ DNA hairpin structures as precursor units, allowing the triggered formation of the four functional strands assembling the CDN X, Figure 4A. The two hairpins H_AA_′ and H_BB_′ include caged sequences of components A, A′, B, and B′. Photochemical (λ = 365 nm) uncaging of the hairpins yields the components A, A′, B, and B′ that are pre-engineered to include each AD-aptamer subunit tethered to a DNA sequence that includes a different Mg^2+^-ion-DNAzyme subunit (different binding arms for the substrate). In the presence of AD and ADA, each of the four components evolves four constituents stabilized by AD into the AD-stabilized CDN X, where each of the constituents is functionalized with a different Mg^2+^-ion-DNAzyme reporter unit that monitors quantitatively the temporal contents of the constituents. The concomitant ADA-catalyzed transformation of AD to inosine brings the CDN X back to the parent reaction module, leading to the phototriggered evolution of the AD-stabilized CDN being transiently depleted. Figure 4B displays the transient temporal concentrations of the constituents in CDN X in the presence of two different concentrations of AD. As the concentration of AD increases, the separation of the constituents and the transient lifetime of the parent reaction module are prolonged. Of course, the corresponding dynamic concentration changes of the constituents in CDN X were computationally simulated by a kinetic model. The dynamic kinetic behavior of the triggered transient CDN X is able to be predicted as the concentration of trigger (AD) changed.

### Operation of Transient Dynamic Circuits and Constitutional Dynamic Networks within Liposome Containments

The AD-triggered dynamic reaction circuits discussed so far were integrated in liposome assemblies, and AD was used as an auxiliary external trigger to operate the dynamic, intraliposome circuits as protocell models. (For details on preparing the loaded liposomes, see Figure S18 and experimental details provided in the Supporting Information.) The respective reaction circuits and networks integrated in giant liposomes (∼16 μm, see Figure S19) were prepared according to the reported procedure.^63^ Figure 5A displays the integration of the two duplexes L_1_F and L_2_Q that include the AD aptamer subunits (a_1_, a_2_) conjugated with the FAM-modified strand F and the BHQ1-modified strand Q. The ADA biocatalyst, as an auxiliary agent, was also included in the liposome units. The average concentration of the circuit constituents of the reaction module in the liposomes corresponded to 0.98 μM per liposome and ADA 0.049 U/mL per liposome. (The concentrations of components were determined by evaluating the concentration of the constituents in the bulk solution before and after preparation of the liposomes. Total concentration of liposomes is 1 × 10^6^ liposomes/mL. The circuit-loaded liposome is subjected to external AD as a trigger. Permeation of AD across the liposome boundary activates the dynamic transient assembly of the AD-stabilized supramolecular assembly composed of L_1_F and L_2_Q. The FRET-induced quenching of F by the spatial proximity of Q within the supramolecular product proceeds. The concomitant ADA-catalyzed transformation of AD to inosine separates the intermediate complex, leading to the recovery of the parent reaction module within the liposome containment. The transient fluorescence intensities associated with the formation/depletion of the supramolecular intermediate in the liposomes, in the presence of different concentrations of the fueling AD and ADA are displayed in Figure 5B,C. Increasing the concentration of exterior AD results in higher permeation of the triggering stimuli and elevated contents of the temporally generated AD/(L_1_F + L_2_Q) complex. Also, increasing the concentration of ADA enhances the transient recovery of the intermediate supramolecular complex to the parent reaction circuit within the liposome containment. Moreover, the transient formation/depletion of the supramolecular AD/(L_1_F + L_2_Q) complex could be followed by temporal confocal microscopy imaging of the liposomes, Figure 5D. The “Rest” liposomes show the green fluorescence nonquenched configuration of the separated L_1_F and L_2_Q duplexes. Subjecting the loaded liposomes to AD results in an immediate quenching of the fluorescent liposomes, consistent with the formation of the quenched supramolecular intermediate AD/(L_1_F + L_2_Q) complex loaded within liposomes. Subsequently, temporal recovery of the fluorescent liposomes is observed due to the ADA-induced separation of the complex. Figure 5D, Panel II depicts the integrated transient fluorescence intensities of the liposomes imaged in the frames, normalized to the number of liposomes, at time intervals of the dissipative process proceeding in the liposome (total of five frames at each time interval, error bars derived from three independent experiments). The temporal integrated fluorescence intensities reveal a transient pattern, where the original fluorescence associated with liposomes is recovered after ca. 400 min. These results are consistent with the dynamic fluorescence changes shown in Figure 5B, curve a.

**Figure 5:**
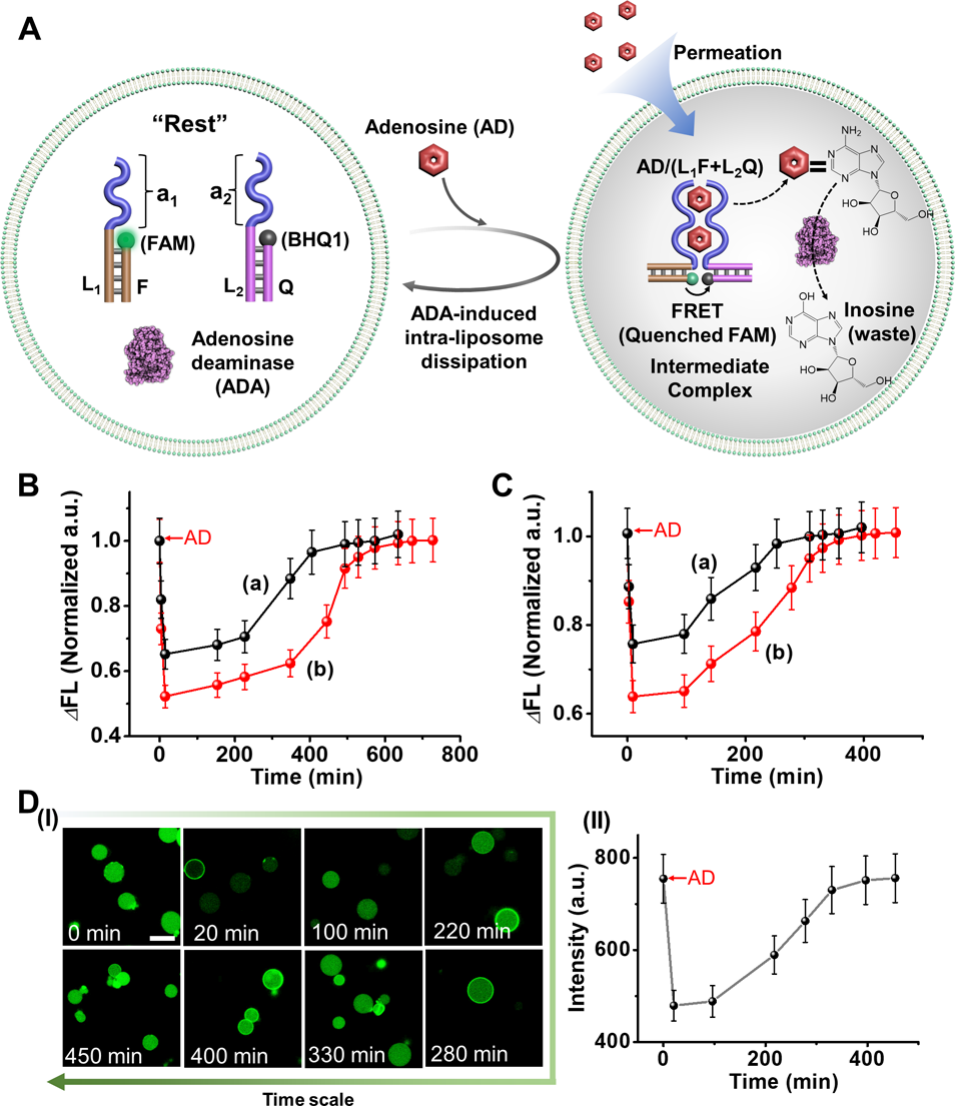
(A) Schematic loading of liposome containments with a reaction module consisting of L_1_F, L_2_Q duplexes, and ADA, and the AD-triggered generation of a transient AD-stabilized intermediate. The temporal intensities of the FRET fluorescence signals generated by the intermediate supramolecular complex probe the dynamic dissipative operation of the system. (B) Temporal transient fluorescence intensity changes in the liposomes in the presence of ADA 0.049 U/mL per liposome using (a) AD = 1 mM, (b) AD = 2 mM. (C) Temporal transient fluorescence intensity changes in the liposomes in the presence of ADA 0.074 U/mL per liposome using (a) AD = 1 mM, (b) AD = 2 mM. (D) Panel I-Time-dependent confocal fluorescence microscopy images of the liposome undergoing transient formation and depletion of the AD (1 mM)-stabilized supramolecular complex in the presence of ADA 0.049 U/mL per liposome. Scale bar: 20 μm. Panel II-Integrated fluorescence intensities normalized to the number of cells in the image frames along the temporal formation and depletion of the supramolecular AD/AD-aptamer subunit complex according to (A). Error bars are derived from three independent experiments where each data point represents the average integrated fluorescence intensities of five frames recorded for each time interval.

The successful integration of the transient AD/ADA-driven supramolecular circuit in liposome assemblies was followed by the integration of functional AD/ADA dynamic DNAzyme circuits and networks in liposome frameworks as simple protocell assemblies. Figure 6A depicts the integration of the photoresponsive unit-caged hairpin H_D1D2_, ADA and the hairpin H_D1D2_, ADA and the reporter substrates were integrated in the liposomes. The photocleavage (λ = 365 nm) of the hairpin H_D1D2_ evolving the two strands D_1_ and D_2_ was in the liposome containment, each 0.49 μM per liposome. Each of the strands includes the AD aptamer subunits a_1_, a_2_, tethered to the Mg^2+^-ion-dependent DNAzyme subunits, p, and q. The fluorophore F_i_ (ROX)/quencher Q_i_ (BHQ1) substrate and ADA were also loaded in the liposomes. (For the homogeneous loading of the DNAzyme constituents in the liposomes, see the Experimental Section, Supporting Information.) The loaded liposomes did not reveal any Mg^2+^-ion-dependent DNAzyme, Figure 6B, Panel I, curve (i). Subjecting the liposomes to AD results in the permeation of AD into the liposomes and the self-assembly of the AD-stabilized aptamer subunit complex that allosterically stabilizes DNAzyme subunits, catalyzing the cleavage of the substrate and the generation of the fluorescence of the fragmented fluorophore-modified substrate. This is reflected by a rapid time-dependent cleavage of the substrate, Figure 6B, curve (ii). Subsequently, a decrease in the rates of cleavage of the substrate by the DNAzyme is observed, Figure 6B, Panel I, curves (iii-iv), and after a time interval of 450 min, the catalytic activities of the DNAzyme are entirely depleted, curve (v). These results are consistent with the fact that AD triggers DNAzyme functions by forming the AD aptamer-subunit complex, accompanied by the concomitant ADA-induced transformation of AD to inosine and separation of the aptamer subunits, therefore depleting the catalytic functions of the DNAzyme. Figure 6B, Panel II, depicts the temporal catalytic rates associated with the AD/ADA-driven formation of the Mg^2+^-ion-DNAzyme. The temporal catalytic rates displayed in panel II (nM/min) were evaluated by translating the fluorescence change rates associated with the supramolecular DNAzyme activities at different time intervals of the transient process into temporal catalytic rates of the DNAzyme complex (nM/min) using the calibration curve shown in Figure S7. The catalytic rates display a transient behavior that follows the AD-stimulated formation of the DNAzyme and the concomitant ADA-induced depletion of the DNAzyme biocatalyst. The transient activities of the DNAzyme are further suggested by temporal fluorescence confocal microscopy imaging of the liposomes, Figure 6C, Panel I. At time *t* = 0 min, no fluorescence of the liposomes is observed. Subjecting the liposomes to AD results in a temporal fluorescence intensity increase in the liposomes. The temporal fluorescence intensity changes of the liposomes, normalized with respect to the number of liposomes imaged in the respective frames, are depicted in Panel II. (Error bars derived from 5 different frames in 3 independent experiments, total 15 frames). A temporal fluorescence saturation after ca. 400 min is observed, indicating the blockage of the DNAzyme functions in the liposomes after this time interval. Figure 6B, Panel III depicts the temporal catalytic rates of the DNAzyme in the liposome containments (first derivative of the curve depicted in Panel II). A transient evolution and depletion of the DNAzyme catalytic rates is observed, demonstrating the transient depletion of the catalytic rates after ca. 400 min, consistent with the results presented in Figure 6B, Panel II. (It should be noted that as AD fuels diffuse into the liposome, inosine diffuses freely through the liposome boundary to the bulk solution. In fact, the generated inosine does not affect the intraliposome transient functions of the DNAzyme, reflected by the recyclability of operating the DNAzyme functions, cf. Figure S20.)

**Figure 6:**
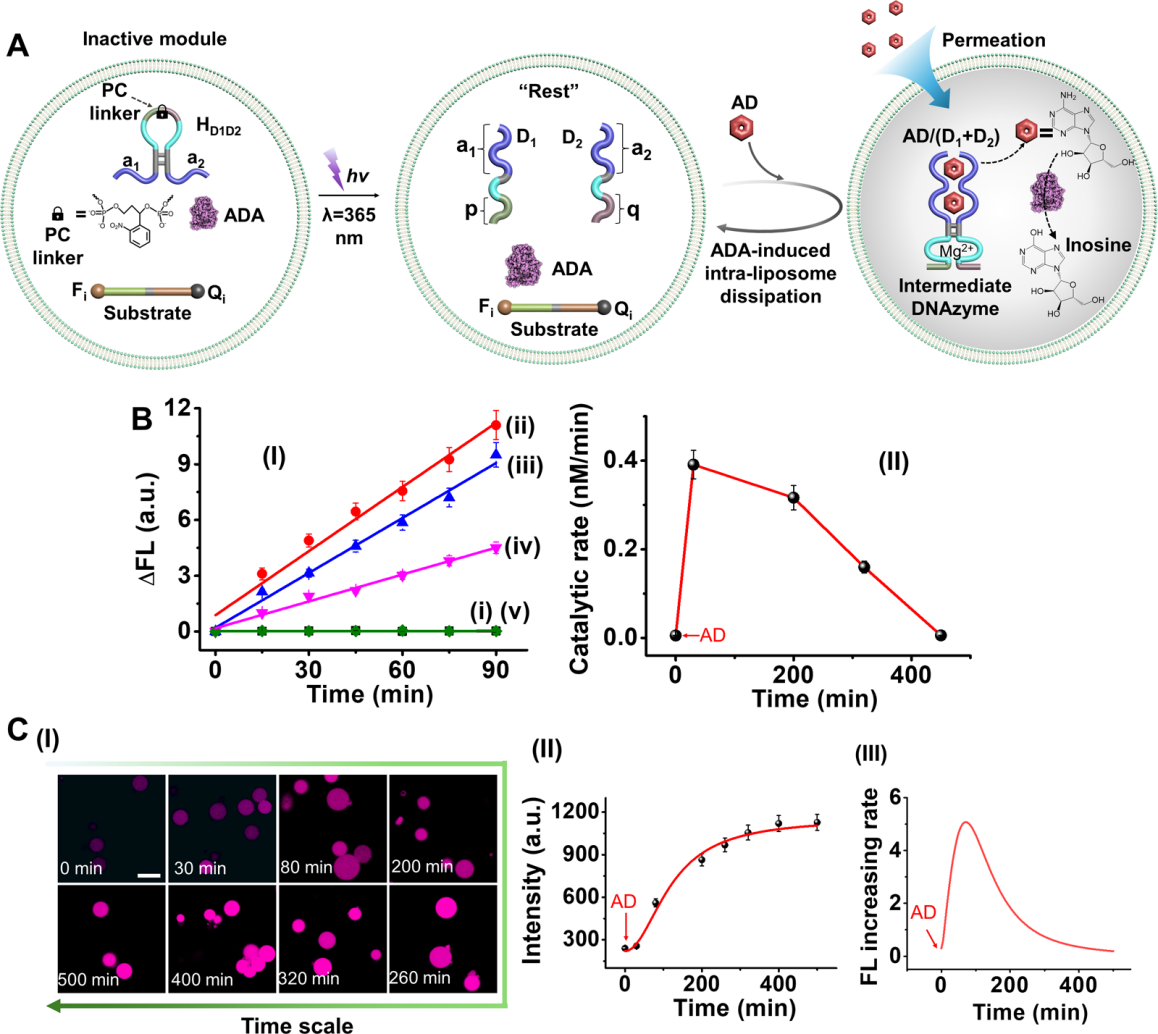
(A) Schematic light-activated, AD-triggered activation of hairpins (H_D1D2_) system in liposome, leading to the transient assembly of an AD-stabilized Mg^2+^-ion-dependent DNAzyme system in the presence of ADA. The transient operation of the DNAzyme are probed by the DNAzyme-catalyzed cleavage rates of the F_i_ (ROX)/Q_i_ (BHQ1)-modified substrate. (B) Panel I-Time-dependent fluorescence change generated at time intervals by the transiently operating DNAzyme reaction module integrated in the liposome assembly. The fluorescence of the liposomes is autozero prior to each time interval of measurement: (i–v) *t* = 0, 30, 200, 300, and 450 min. Panel II: transient catalytic rates corresponding to the cleavage of the F_i_/Q_i_-substrate at time intervals of the DNAzyme operation. Error bars are derived from three independent experiments. (C) Panel I-Confocal fluorescence microscopy images at time intervals of the AD-triggered transient AD-aptamer subunits supramolecular DNAzyme-driven cleavage of the F_i_/Q_i_ substrate shown in (A). Scale bar: 20 μm. Panel II-Transient integrated fluorescence intensities of temporally generated fluorescent liposomes, normalized to the number of liposomes in the imaged frames. Each data point represents an analysis of 5 frames at the respective time intervals. Error bars were derived from three independent experiments. Panel III-Temporal catalytic rates corresponding to the cleavage of the substrate F_i_/Q_i_ monitored by the time-dependent confocal microscopy images (first-order derivative of the temporal fluorescence intensity changes shown in Panel II).

In addition, the transient AD-stabilized CDN was integrated in liposome containments, as shown in Figure 7. Toward this goal, the photoresponsive unit-caged hairpins H_AA_′ and H_BB_′, ADA and the respective reporter substrates (vide infra) were integrated in the liposomes, whereafter the photocleavage (λ = 365 nm) of the hairpins evolved the four components (A, A′, B, B′) as a “Rest” reaction module, Figure 7A. Subjecting the loaded liposomes to auxiliary AD as fuel and permeation of AD across the boundary of the liposome evolved the function CDN X composed of the four AD-stabilized constituents (AA′, AB′, BA′, and BB′). The ADA present in the liposome containments catalyzes the transformation of AD to inosine, resulting in the depletion of the CDN X to the parent “Rest” reaction module and a transient dissipative operation of CDN X in the liposomes. (For the homogeneous loading of the constituents of CDN X in the liposomes, see the Experimental Section, Supporting Information.) The constituents comprising CDN X include each a different Mg^2+^-ion-dependent DNAzymes (different “arms”) as reporter units that probe quantitatively the temporal concentrations of the constituents during the dynamic operation of the CDN. (To analyze the temporal concentrations of the constituents and to minimize perturbances in the analysis, four samples of the CDN X/ADA-loaded liposomes were prepared in the same experimental process. Each of these samples was loaded with a mixture of the respective F_*x*_/Q_*x*_-functionalized substrate analyzing a respective constituent and comixed, nonlabeled substrates, being cleaved by the other DNAzyme reporters without generating any fluorescence.) Photocleavage of the hairpins and their triggering by AD generated the transient CDN X in the samples, allowing the “clean” temporal analysis of the cleavage rates of the F_*x*_/Q_*x*_-functionalized substrates. The transient temporal catalytic rates associated with each of the constituents were evaluated and displayed in Figure 7B. (Time-dependent fluorescence change generated at time intervals by the transiently operating CDN X integrated in the liposome assembly, see Figure S21.) The ADA-catalyzed depletion of CDN X, generating the “Rest” reaction module, is completed within ca. 400 min. Moreover, confocal microscopy imaging of the reaction samples at time intervals of the dissipative formation and depletion of CDN X is displayed in Figure 7C, Panel I_a–d_. Each of the samples demonstrates a time-dependent increase in the integrated fluorescence intensities (normalized to the number of liposomes in the respective frames) that reach saturation levels after ca. 400 min. (It is worth noting that the ratio of constituent concentration to substrate concentration in the system is 1:10, which can maintain a linear slope within 800 min.) The temporal fluorescence changes and their first-order derivatives demonstrate, as expected, transient catalytic rates for the dynamic formation and separation (depletion) of CDN X, Figure 7C, Panels II_a–d_ and III_a–d_.

**Figure 7:**
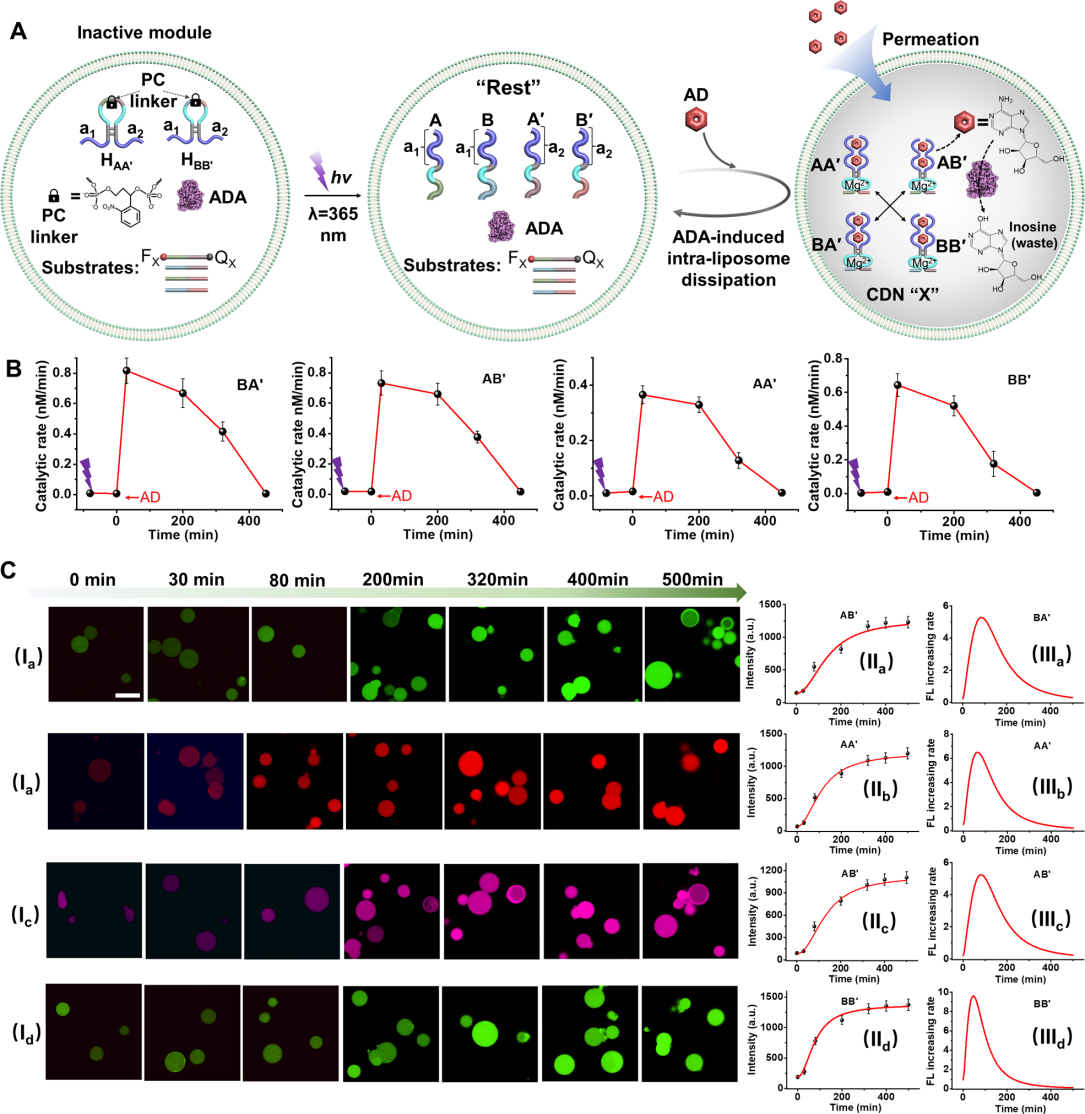
(A) Schematic light-activated, AD-triggered, reaction module consisting of photoresponsive H_AA_′, H_BB_′ hairpins, in the presence of ADA, yielding transiently operating AD-stabilized CDN. The transient content of the CDN constituents is probed following the cleavage rates of the respective F_*x*_/Q_*x*_-modified substrates by the Mg^2+^-ion-reporter units tethered to the respective constituents. (B) Transient catalytic rates corresponding to the cleavage of the F_*x*_/Q_*x*_-substrate at time intervals of the AD/ADA-driven operation of CDN X. Error bars are derived from three independent experiments. (C) Panel I-Confocal fluorescence microscopy images at time intervals of cleavage of the fluorophore/quencher-modified substrates associated with the constituents AA′, BB′, AB′, and BA′ in samples withdrawn from the AD-triggered transient CDN X reaction module in liposome. Scale bar: 20 μm (I_a_–I_d_) corresponding to BA′, AB′, AA′, and BB′ constituents, respectively. Panel II-Transient integrated fluorescence intensities of temporally generated fluorescent liposome, normalized to the number of liposomes in the imaged frames. Each data point represents analysis of 5 frames at the respective time interval. Error bars derived from three independent experiments. (II_a_–II_d_) corresponding to BA′, AB′, AA′, and BB′ constituents, respectively. Panel III-Temporal catalytic rates corresponding to the cleavage of the F_*x*_/Q_*x*_-modified substrate monitored by the time-dependent confocal microscopy images (first-order derivative of the temporal fluorescence intensity changes shown in Panel II). (III_a_–III_d_) corresponding to BA′, AB′, AA′, and BB′ constituents, respectively.

### Liposomes Loaded with Allosterically ATP-Stabilized AD-Aptamer Subunits DNAzymes for Selective Gene Therapy

Our study demonstrated the capacity to evolve an allosterically AD-stabilized DNAzyme framework in liposome containments through the light-triggered cleavage of an o-nitrobenzyl phosphate caged hairpin structure. Moreover, the study emphasized the selectivity of the AD-guided formation of the AD-aptamer subunit-stabilized DNAzyme in comparison to other nucleotides yet demonstrated that other adenine-containing ligands, such as ATP, could substitute AD in allosteric activation of the DNAzyme. With these pieces of information, we searched for potential medical applications of allosterically ATP-stabilized DNAzymes encapsulated in liposome containments. In this subsection, we address the use of allosterically ATP-stabilized DNAzymes, encapsulated in liposomes, as functional carriers for the selective gene therapy of cancer cells. The concept to reach these goals is displayed in Figure 8. Liposomes loaded with a photoresponsive caged hairpin H_EE**′**_ are used as functional carriers to induce the gene therapy process. The fusion of the loaded liposomes with the cancer cells delivers the hairpin-loads into the cancer cells. The hairpin structure H_EE′_ is, however, pre-engineered to evolve upon photocleavage (λ = 365 nm) two strands E, E′ consisting of AD aptamer subunits conjugated to the Mg^2+^-ion-DNAzyme subunits cleaving the EGR-1 mRNA present in the cells. ATP (overexpressed in cancer cells) evolves, and then the allosterically ATP-stabilized DNAzyme cleaves the EGR-1 mRNA, leading to the apoptosis of the cancer cells. The development of the system requires, however, fusion of the liposomes with the cells and delivery of the loads into the cells. Toward this goal, the liposomes are functionalized with the cholesterol-modified DNA strand x, and the cells are functionalized with the strand x′ (complementary to x) conjugated to the AS1411 aptamer unit that binds to the nucleolin receptors associated with the cancer cells. The hybridization between x/x′ leads to the fusion of the liposomes with the cells,^64^ resulting in the delivery of the hairpin load into the cells. Note that the duplex of the x/x′-AS1411 aptamer is dictated by the nucleolin receptor sites associated with the cancer cells, resulting in selectivity in delivery and mRNA cleavage efficacy in the cancer cells. (For a detailed explanation on the selective cleavage of the EGR-1 mRNA by the H_EE′_-loaded liposomes, see Supporting Information pages S31–S34). To develop the system and demonstrate the selectivity of treatment, two cell lines were employed. These include MCF-7 breast cancer cells, which include the nucleolin receptor units in their cell membrane boundaries, and MCF-10A epithelial breast cells lacking the nucleolin receptor in their cell membranes. The characterization of the system included two steps: (i) Characterization of the fusion process between the liposomes and the cell lines. (ii) The evaluation of the allosterically ATP-stabilized DNAzyme activity in the cells and the evaluation of the EGR-1 mRNA cleavage and accompanied apoptosis in the respective cells.

**Figure 8:**
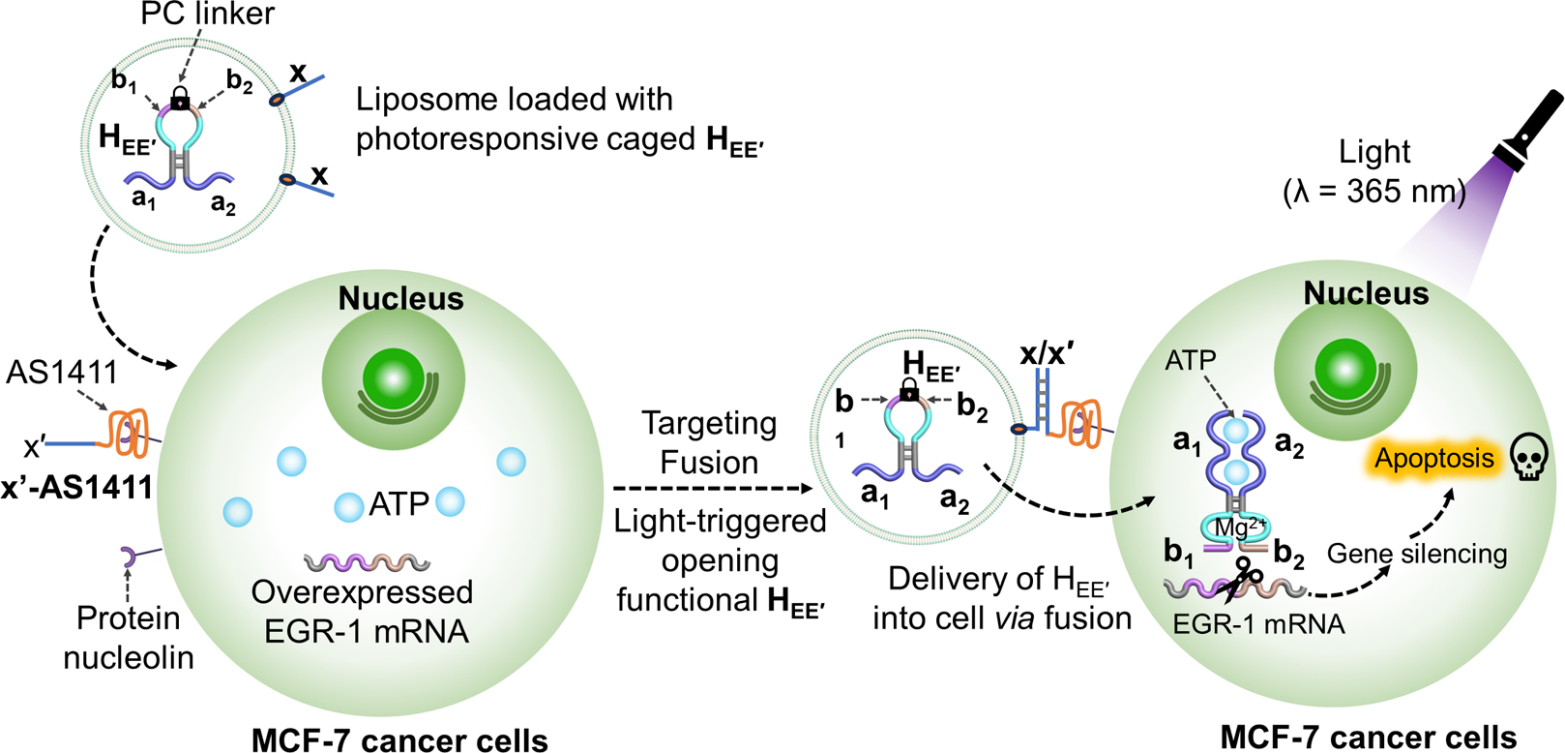
Schematic fusion of tether x-modified liposome loaded ATP-responsive light-activated hairpin H_EE′_ into x′-AS1411-functionalized MCF-7 cancer cells leading to selective cell apoptosis by the light-activated intracellular overexpressed ATP-assembled DNAzyme cleaving EGR-1 mRNA (the hairpins H_EE′_ include AD-aptamer subunits a_1_, a_2_, and EGR-1 mRNA binding arms b_1_, b_2_.).

In the first step, liposomes loaded with a fluorescent reaction module, lacking the cholesterol-modified x-tethers or modified with the cholesterol-functionalized x-tethers, were interacted with MCF-7 or MCF-10A cells treated with the x′-AS1411 aptamer-conjugated strands to probe the fusion process between loaded liposomes and cells, and the delivery of the fluorescent probe into the cells. The liposome-cell fusion processes and the delivery of loads are presented in Figures S22 and S23. The results of these experiments are discussed in detail in the Supporting Information, pages S32 and S33. The main conclusion of these experiments is (i) The bridging between the liposomes and MCF-7 cells by means of the x/x′-AS1411 aptamer hybrid enhanced the fusion process and the delivery of the loads into the MCF-7 cell cytoplasm. (ii) Selective fusion of the x-strand-modified liposomes with the MCF-7 cells is demonstrated, and no fusion with the MCF-10A cells is observed due to lack of the nucleolin receptors in these cells. In the next step, the cleavage efficacy of the EGR-1 mRNA by liposomes delivering the DNAzyme constituents that cleave the mRNA was evaluated. The two cell-lines, MCF-7 or MCF-10A, treated with two types of liposomes delivering the functional DNAzyme, are presented in Figure 9A. One kind of x-strand-modified liposomes (configuration I) was loaded with the photoresponsive caged hairpin H_EE′_, which was subsequently photochemically uncaged (λ = 365 nm) to yield the separated strand E, E′, Figure 9A, Panel I. The separated E/E′-loaded liposomes were fused with the x′-AS1411 aptamer-modified MCF-7 cells, resulting in the delivery of E and E′ strands into the cell cytoplasm. The intracell-loaded strands undergo self-assembly into the allosterically ATP-stabilized DNAzyme, resulting in the cleavage of EGR-1 mRNA and cell apoptosis. The second type of x-strand-modified liposomes (configuration II) was loaded with the caged H_EE′_ hairpin, fused with the x′-AS1411 aptamer-modified MCF-7 cells, and the load was delivered to the cell cytoplasm, Figure 9A, Panel II. The light-activated intracellular separation of H_EE′_ (λ = 365 nm) resulted in the formation of the allosterically ATP-stabilized DNAzyme supramolecular complex, cleaving the EGR-1 mRNA and cell apoptosis. Parallel experiments treating the MCF-10A epithelial cells with the x′-AS1411 aptamer and their subsequent interaction with the two kinds of liposomes were examined as a control. (For experimental details of the fusion processes and additional control experiments, see Supporting Information, pages S33 and S34). Figure 9B depicts the EGR-1 mRNA cleavage-induced cell apoptosis stimulated by the two kinds of loaded liposomes on the MCF-7 and MCF-10A, reflected by the cell viabilities. (The results for a set of control systems are also provided.) Evidently, the MCF-7 cells treated with liposomes (configuration I) shown in Figure 9A, Panel I, revealed a cell apoptosis of ca. 75%, while the MCF-10A cells were unaffected, entry (a). Similarly, MCF-7 treated with liposomes (configuration II) shown in Figure 9A, Panel II, demonstrated a cell apoptosis of ca. 80%, while the MCF-10A epithelial cells were unaffected. The results demonstrate effective and selective apoptosis of the MCF-7 cells, consistent with the effective x/x′-AS1411-induced fusion of the loaded liposomes with the MCF-7 cells, activating the allosterically ATP-stabilized DNAzyme, catalyzing the cleavage of EGR-1 mRNA and subsequent cell apoptosis. The remarkable selectivity, which spares the MCF-10A epithelial cells, is due to the absence of nucleolin receptors, preventing the fusion process from occurring.

**Figure 9:**
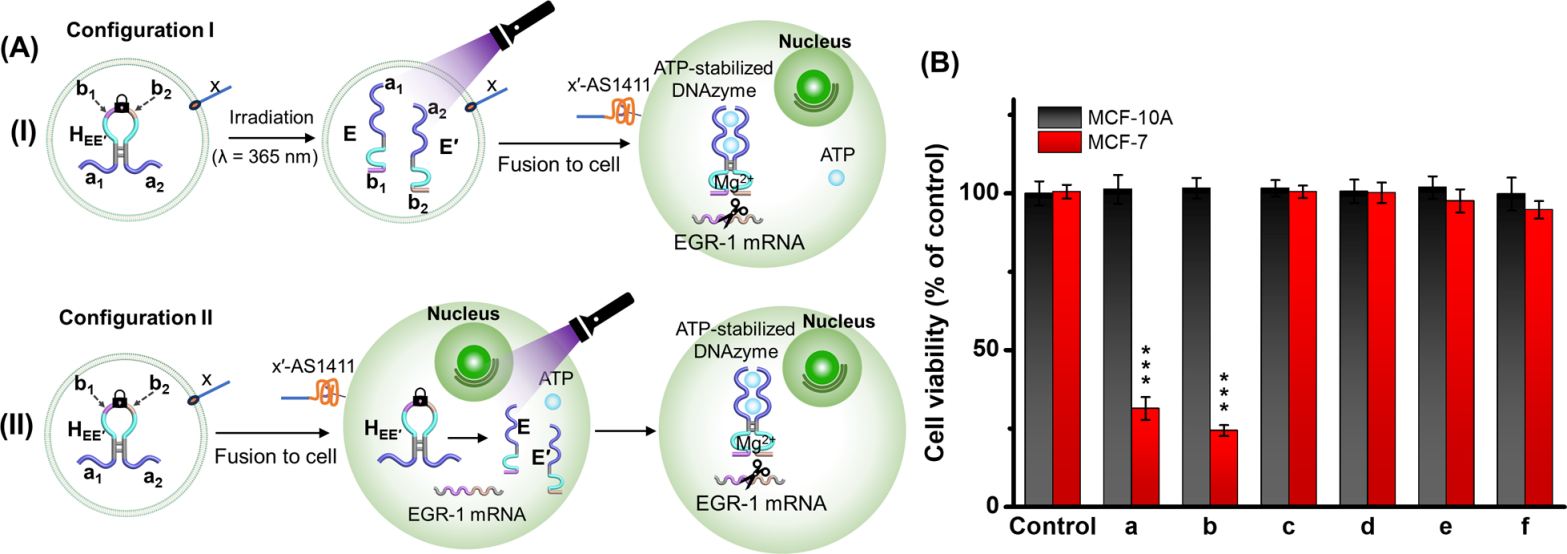
(A) Panel I-Configuration I: Preirradiated (λ = 365 nm) liposomes where the hairpins H_EE′_ were separated into strands E, E′ prior to fusion and delivery into the cells. Panel II-Configuration II: Fusion of caged hairpins H_EE′_-loaded liposomes with cell contaminants enables delivery of caged hairpins H_EE′_ into cell containments. Subsequent irradiation (λ = 365 nm) induces release of strands E and E′ in cells. (B) Relative cell viability of MCF-10A cells and MCF-7 cells subjected to the different loaded liposomes: (a) tether x-modified liposomes loaded with the preirradiated hairpins H_EE′_; (b) tether x-modified liposomes loaded with the caged hairpin H_EE′_ subsequently being intracellularly activated by light (λ = 365 nm); (c) nonirradiated and nontether x-modified liposomes loaded with the caged hairpins H_EE′_; (d) nonirradiated and tether x-modified liposomes loaded with the caged hairpins H_EE′_; (e) nontether x-modified liposomes loaded with the caged hairpins H_EE′_ treated with preirradiation by light (λ = 365 nm). (f) nontether x-modified liposomes loaded with the caged hairpin H_EE′_ subsequently being intracellularly activated by light (λ = 365 nm). Note: All the cells (MCF-10A and MCF-7) were incubated for 4 h upon subjecting the different loaded liposomes to cells. After washing with growth medium, cells were replenished with full medium and further incubated for 24 h. Error bars derived by analyzing *N* = 4 frames of cells. Significant results were evaluated using the *t*-test; ***P < 0.001.

## Conclusions

The study introduced the application of an auxiliary ligand (adenosine, AD) and a conjugated ligand depleting enzyme (ADA) as triggers to assemble functional aptamer-subunit-based constitutional dynamic networks and circuits exhibiting dynamic reconfiguration and transient operation features. This is a versatile concept that can be extended to other ligand/enzyme depleting systems such as acetylcholine/acetylcholine esterase and peptides/proteases in the presence of the respective aptamer-subunit ligand constituents. Moreover, the integration of these ligand/aptamer subunits and enzymes in liposome containment has been presented, and the dynamic operation of the networks and circuits in these cell-like containments, as biomimetic protocell assemblies, was demonstrated. A versatile method for assembling the dynamic network in the protocell assemblies was introduced. This included the loading of photoresponsive caged hairpins in the liposome containments and their photochemical uncaging into multicomponent units self-assembling into complex networks and circuits.

The systems and results spark significant potential advancement of the area of Systems Chemistry by synthetic dynamic DNA networks and circuits embedded in liposome containments and particularly introducing visionary applications of such systems. Enhancing the complexity of the synthetic network/circuit-loaded liposome containments by engineering the loaded constituents and liposome structures and their functionalities could be an interesting path to follow. This could include the functionalization of the liposome boundaries with specific pore transporting channels and specifically with stimuli-responsive gated channels, allowing signal-triggered activation of the liposome-loaded synthetic circuits. Alternatively, increasing the complexity of synthetic networks and circuits loaded in the liposome containments represents an important route to follow. Designing of multidimensional dynamic synthetic networks, bistable/orthogonal and oscillatory dynamic circuits, and cascaded and feedback-driven circuits in the liposome frameworks represents challenging goals for future protocell systems development. In addition, the self-assembly of cell-like subcontainments or synthetic organelles guiding stimuli-responsive functionalities can be envisaged.

Moreover, our study demonstrated the successful fusion of pre-engineered photoresponsive caged hairpin-loaded liposomes with cancer cells as a functional platform for selective gene therapy. Specifically, we demonstrated the dictated fusion of pre-engineered hairpin-loaded liposomes with cancer cells, leading to selective intracellular photoactivated allosterically ATP-stabilized DNAzyme units catalyzing the EGR-1 mRNA cleavage and subsequent apoptosis of the cancer cells. In principle, the concepts developed in this study could be implemented to develop many other gene therapy pathways. For example, the engineering of photoactivated DNAzyme structures being allosterically stabilized by other cell biomarkers, e.g., miRNA, could lead to the cleavage of mRNAs related to diverse other diseases. In addition, the selectivity induced by the x/x′-aptamer liposome-cell fusion mechanism could be applied for delivering many other synthetic networks or DNA machineries, e.g., CRISPR-Cas12a for gene therapy or gene editing. While the UV-light deprotection of the systems might be a drawback, the application of modified photoresponsive caging units^62^ or the use of upconversion particles^65^ could shift the photodeprotection process to the visible or even near-infrared spectral range for practical applications.

## Supplementary Material



## Notes

All detailed experimental methods, simulation model, calibration curves, and size distribution of loaded liposomes (PDF)
