# Supplementary material for: Adenosine-Triggered Dynamic and Transient Aptamer-Based Networks Integrated in Liposome Protocell Assemblies
Source: J Am Chem Soc. 2025 May 22;147(22):19282–95. doi: 10.1021/jacs.5c05090 (PMC12147132; doi:10.1021/jacs.5c05090)
Supplement: Supplementary file 1 [file ja5c05090_si_001.pdf]

Supporting Information

Adenosine-Triggered Dynamic and Transient Aptamer-Based Networks Integrated in Liposome Protocell Assemblies

Yu Ouyang,[a] Yang Sung Sohn,[c] Xinghua Chen,[a] Rachel Nechushtai,[c] Eli Pikarsky,[d] Fan Xia,[b] Fujian Huang,[b]* and Itamar Willner[a]*

[a] Institute of Chemistry, The Hebrew University of Jerusalem, Jerusalem 91904, Israel
[b] State Key Laboratory of Geomicrobiology and Environmental Changes, Faculty of Materials Science and Chemistry, China University of Geosciences, Wuhan 430074, China
[c] Institute of Life Science, The Hebrew University of Jerusalem, Jerusalem 91904, Israel
[d] The Lautenberg Center for Immunology and Cancer Research, IMRIC, The Hebrew University of Jerusalem, Jerusalem 91120, Israel

*Email: itamar.willner@mail.huji.ac.il; huangfj@cug.edu.cn

Materials and Methods

Materials

Adenosine (AD), Trizma® base, magnesium chloride hexahydrate, adenosine Deaminase (ADA) and cholesterol were purchased from Sigma-Aldrich. 1,2-di-(9Z-octadecenoyl)-sn-glycero-3-phosphocholine (DOPC, Pro. No. 850375P), 1,2-dioleoyl-sn-glycero3-phosphoethanolamine (DOPE), and cholesterol were purchased from Sigma-Aldrich. (3-Aminopropyl)trimethoxysilane (APTES) coated slides were purchased from Electron Microscopy Sciences (Hatfield, PA). Acrylamide/bisacrylamide solution (40%, 37.5:1) was acquired from Bio-Lab (Jerusalem, Israel). Glass bottom well plates for confocal imaging were acquired from Cellvis (Mountain View, CA).

The nucleic acid sequences used in the study were custom ordered (Integrated DNA Technologies, IDT), and the respective sequuences were:

L_1: 5′- ATAACGCATT T TGTTA **TGCGGAGGAAGGT**AT-3′
L_1′: 5′- AATTCTAATAACGCATTTTGTTA **TGCGGAGGAAGGT**AT-3′
L_2: 5′-AT**ACCTGGGGGAGTAT**TAACAT GTTCCATCTT-3′
L_2′: 5′-AT**ACCTGGGGGAGTAT**TAACATGTTCCATCTTGATTTAA-3′
GTTCCATCTTGATTTAA
F: 5′-/56-FAM/AATGCGTTATTAGAATT -3′
F′: 5′-/Cy3/AATGCGTTATTAGAATT-3′
Q: 5′-TTAAATCAAGATGGAAC/3BHQ_1/-3′
P: 5′-TTAAATCAAGATGGAAC-3′
D_1: 5′-GTCCTC AGCGAT TACTA **TGCGGAGGAAGGT**AT-3′
D_2: 5′-AT**ACCTGGGGGAGTAT** TAGTA CACCCATGT TCCTGA-3′
H_{D1D2}: 5′-AT**ACCTGGGGGAGTAT** TAGTA CACCCATGT TCCTGA/iSpPC/
GTCCTC AGCGAT TACTA **TGCGGAGGAAGGT**AT-3′
A: 5′-GTCCTC AGCGAT TACTA **TGCGGAGGAAGGT**AT-3′
A′: 5′-AT**ACCTGGGGGAGTAT** TAGTA CACCCATGT TTCAGT-3′
B: 5′-CTGTTC AGCGAT TACTA **TGCGGAGGAAGGT**AT-3′
B: 5′-AT**ACCTGGGGGAGTAT** TAGTA CACCCATGT TCCTGA-3′
$H_{AA'}$:5′-AT**ACCTGGGGGAGTAT** TAGTA CACCCATGT TTCAGT /iSpPC/
GTCCTC AGCGAT TACTA **TGCGGAGGAAGGT**AT-3′
$H_{BB'}$: 5′-AT**ACCTGGGGGAGTAT** TAGTA CACCCATGT TCCTGA /iSpPC/
CTGTTC AGCGAT TACTA **TGCGGAGGAAGGT**AT-3′
Sub1 (BA′): 5′-FAM-ACTGAATrAGGAACAG-BHQ1-3′
Sub2 (AB′, D_1D_2): 5′-ROX-TCAGGATrAGGAGGAC-BHQ2-3′
Sub3 (AA′): 5′-Cy5-ACTGAATrAGGAGGAC-BHQ2-3′
Sub4 (BB′): 5′-FAM-TCAGGATrAGGAACAG-BHQ1-3′
Sub1-noFQ (BA′): 5′-ACTGAATrAGGAACAG-3′
Sub2-noFQ (AB′, D_1D_2): 5′-TCAGGATrAGGAGGAC-3′
Sub3-noFQ (AA′): 5′-ACTGAATrAGGAGGAC-3′
Sub4-noFQ (BB′): 5′-TCAGGATrAGGAACAG-3′
Inhibitor: 5′-ATACTCCCCCAGGTAT-3′
x: 5′-/Chol-TEG/TTTTTT*ACCTGGGGGAGTATTGGATA*CACCCATGTTCCTGA-3′
x′-aptamer AS1411:5′-
*TATCCAATACTCCCCCAGGT*TTTGGTGGTGGTGGTTGTGGTGGTGGTG
G-3′

H_{EE}': 5′-ATACCTGGGGGAGTATTAACAGACGAGCA/iSpPC/
CCGCGGCCAGGCTAGCTACAACGACCTG TGTTA TGCGGAGGAAGGTAT-3′

Instrumentation

Absorption spectra were recorded at 25 °C using a UV-2450 spectrophotometer (Shimadzu), a cuvette of 50 μL volume (made of quartz suprasil, Hellma Analytics) was used in these experiments. Fluorescence spectra were recorded at 25 °C using a Cary Eclipse Fluorometer (Varian Inc) was used in these experiments. The excitation of FAM, ROX, and Cy5 were performed at 496, 588, and 648nm, respectively. The emission of FAM, ROX, and Cy5 were recorded at 516, 608, and 668 nm, respectively. UV light source (fixing the light intensity of 5 mW/cm^2) is carried on THORLABS device including LED (M365LP1-C2) and driver (M00760962).

Methods

Adenosine-triggered ADA-assisted transient, dissipative reaction circuit.

A mixture solution of 0.5 μM duplexes L_1/F, L_2/Q, and 0.025 U/mL ADA in 20 mM Tris-HCl Buffer (20 mM magnesium chloride, pH 7.38@25°C) was incubated at 25 °C for 1 h. The resulting "mute" reaction module solution was transferred into the cuvette and triggered by variable concentrations of AD. The time-dependent fluorescence changes were monitored spectroscopically at 25 °C.

Adenosine-triggered ADA-assisted transient, dissipative DNAzyme circuit.

A large volume (1 mL) mixture solution of 0.5 μM AD-aptamer subunits-modified strands D_1, D_2 and 0.025 U/mL ADA in 20 mM Tris-HCl Buffer (20 mM magnesium chloride, pH 7.38@25°C) was incubated at 25 °C for 1 h. The resulting "mute" reaction module solution was triggered by variable concentrations of AD (3 mM, 4 mM, and 5 mM). Subsequently, aliquots of 30 μL were withdrawn from the solution at different time-intervals and treated with sub2 (5 μM). The time-dependent fluorescence changes were followed at different time-intervals under 25 °C. By using the appropriate calibration curves corresponding to the cleavage rate of the substrate by the intact constituent at various concentrations, the concentrations of the DNAzyme constituent in DNAzyme catalytic circuits within the course of AD-triggered transient DNAzyme circuit were quantified.

Adenosine-triggered ADA-assisted transient evolution of constitutional dynamic network (CDN X).

A large volume (1 mL) mixture solution of a single strand "pool" of A, A′, B, and B′, 1 μM each, and 0.025 U/mL ADA in 20 mM Tris-HCl Buffer (20 mM magnesium chloride, pH 7.38@25°C) was incubated at 25 °C for 1 h. The prepared above "mute" reaction module solution was then treated with variable concentrations of AD (4 mM, 5 mM). Aliquots of 30 μL were withdrawn from the solution at different time-intervals and treated with sub1, sub2-noFQ, sub3-noFQ, and sub4-noFQ (for BA′), or with sub2-noFQ, sub1, sub2-noFQ, and sub4-noFQ (for AB′), or with sub3-noFQ, sub1-noFQ, sub2, and sub4-noFQ (for AA′), or with sub4-noFQ, sub1-noFQ, sub2-noFQ, and sub3

(for BB′), 1.5 μL of 100 μM each substrate. Subsequently, the time-dependent fluorescence changes were followed at different time-intervals under 25 °C. By using the appropriate calibration curves corresponding to the cleavage rates of the different substrates by the intact constituents at various concentrations, the concentrations of the constituents in transient reaction module solution at different time intervals were quantified.

Photochemically triggered Adenosine-induced transient evolution of CDN X.

H_{AA}' and H_{BB}' (50 μM) in 20 mM Tris-HCl Buffer (20 mM magnesium chloride, pH 7.38@25°C) was annealed at 95 °C for 5 min, cooled down quickly to 25 °C in short time and equilibrated at 25 °C for 2 h. Note that, H_{AA}' and H_{BB}' hairpins one contain o-nitrobenzyl phosphate photoresponsive unit that is photo-deprotected at λ = 365 nm. A large volume (1 mL) mixture solution of two hairpin strands of H_{AA}' and H_{BB}', 1 μM each, and 0.025 U/mL ADA in 20 mM Tris-HCl Buffer (20 mM magnesium chloride, pH 7.38@25°C) was incubated at 25 °C for 1 h. The above "inactive" reaction solution was activated first by the light (λ = 365 nm) for 10 min. The activated "mute" reaction module solution was then treated with variable concentrations of AD (4 mM, 5 mM). Aliquots of 30 μL were withdrawn from the solution at different time-intervals and treated with sub1, sub2-noFQ, sub3-noFQ, and sub4-noFQ (for BA′), or with sub2-noFQ, sub1, sub2-noFQ, and sub4-noFQ (for AB′), or with sub3-noFQ, sub1-noFQ, sub2, and sub4-noFQ (for AA′), or with sub4-noFQ, sub1-noFQ, sub2-noFQ, and sub3 (for BB′), 1.5 μL of 100 μM each substrate. Subsequently, the time-dependent fluorescence changes were followed at different time-intervals under 25 °C. By using the appropriate calibration curves corresponding to the cleavage rates of the different substrates by the intact constituents at various concentrations, the concentrations of the constituents in transient reaction module solution at different time intervals were quantified.

Encapsulation of the AD-triggered transient reaction circuit, DNAzyme circuit and evolution of CDN X in liposomes.

Liposomes were prepared using the gel swelling method, in which vesicles are generated from lipid films deposited on a polyacrylamide gel substrate, Figure S1. See below for details, APTES-coated slides were incubated with 10% (v/v) glutaraldehyde for 30 minutes and dried before three-times washing with water. Gel solution was prepared by adding 1 μL tetramethylethylenediamine and 10 μL ammonium persulfate (10% wt/v) to 1 mL of 3.25% acrylamide/bisacrylamide. After a brief vortexing, 20 μL of this solution was applied to glutaraldehyde-functionalized glass slides, and a glass coverslip was placed on top to ensure flattening. The gels were left to polymerize for 30 minutes, after which the glass coverslips were removed. The polyacrylamide gels were then rinsed three times with deionized water and dried at 45 °C for 30 minutes. This process produced thin, flat polyacrylamide gels covalently bonded to the glass slides. Lipid suspensions were prepared at a concentration of 13.5 mM DOPC in a 10 : 1 $CHCl_3$:MeOH mixture. A 20 μL aliquot of the lipid suspension was applied to the

polyacrylamide gels and evenly spread to cover their surfaces. The resulting lipid films on the gel substrates were then dried under vacuum for at least 15 minutes. The 1 mL corresponding loading-inner solutions (the loading-inner solutions in all systems are detailed below) of the AD-triggered reaction solutions were prepared for the following incubation with the lipid-covered glass slide in well-sealed round petri dish overnight at 30 °C.

- Loading-inner solution of AD-triggered transient reaction circuits includes a mixture solution of 1 μM duplexes L_1/F, L_2/Q, and 0.05 U/mL ADA in 20 mM Tris-HCl Buffer (20 mM magnesium chloride, 10 mM sucrose, pH 7.38@25°C).
- Loading-inner solution of AD-triggered transient DNAzyme circuits includes a mixture solution of 0.5 μM AD-aptamer subunits-modified hairpin strands H_{D1D2}, 5 μM Sub-2 and 0.05 U/mL ADA in 20 mM Tris-HCl Buffer (20 mM magnesium chloride, 10 mM sucrose, pH 7.38@25°C).
- Taking sub-1 reporter system as example, the loading-inner solution for AD-triggered transient CDN X circuits consists of a mixture containing 1 μM each of the hairpin strands $H_{AA'}$ and $H_{BB'}$, 5 μM of Sub-1, Sub2-noFQ, Sub3-noFQ, and Sub4-noFQ (for BA′), and 0.005 U/mL ADA enzyme. The solution is prepared in a buffer comprising 20 mM Tris-HCl, 20 mM magnesium chloride, and 10 mM sucrose, with a pH of 7.38 at 25°C. Following the above example, the other substrate reporters (e.g., Sub2, Sub3, and Sub4) system associated with the CDN X loaded in liposome were well-prepared. For the best comparison, all the liposome samples were prepared in same day and same procedure.

The prepared reaction system-loaded liposomes were further washed and purified via the mixed cellulose esters membrane (pore size: 3 μm) by 20 mM Tris-HCl Buffer solution (20 mM magnesium chloride, 10 mM glucose, pH 7.38@25°C) for three times. The loaded liposome solutions were checked by the cell counter and placed in 4 ℃ for further using.

Issues related to the homogeneity of the resulting liposomes

It should be noted that issues related to the homogeneity of the structural features of the liposomes and their compositional loading should be considered:

1. Obviously, the sizes of the fabricated liposomes are in the range of 10-20 μm diameters (cf. Figure S19).
2. The contents of the loads in the different sized liposomes differs, yet the concentrations of the loads in the liposomes are similar, due to the method of synthesizing the loaded liposomes (please see point 3 below.) These mean that the loaded liposomes exhibit load-concentration homogeneity (and “empty” liposomes may be present), the “activity” of the liposomes originates from a collective distribution of functional liposomes exhibiting load-concentration homogeneity.
3. It should be noted that the concentration-homogeneities of the DNAzyme constituents in Figure 6 and of the CDN constituents in CDN X shown in Figure 7 are achieved by employing the photochemical cleavage of the hairpin loads

after their encapsulation in the liposomes. For example, a single hairpin H_{D1D2} at the specific concentration is encapsulated in the liposomes. Although the contents of H_{D1D2} in the different sized liposomes might be different, the concentrations of the hairpins in the different sized liposomes are similar. The photocleavage of the hairpin structures, yielding two separated strands, retains the "concentration homogeneity" of the cleaved strands in the liposomes.

Additional notes related to the preparation of the loaded liposomes (See also Figure S18): As we prepared all the inner reaction solutions including AD-triggered transient circuit, AD-triggered transient DNAzyme circuit, and photo-cleaved AD-triggered CDN X, all inner reaction solutions were separately subjected to the polyacrylamide gel-coating glass slide covered with lipid film for overnight incubation. The phospholipid bilayer of liposome consists of phospholipids that are "amphoteric" (amphiphilic, bipolar). In addition, the phospholipid molecule has a hydrophilic phosphate head, and a hydrophobic tail composed of two chain fatty acids. Therefore, aqueous loading-inner solutions penetrate the hydrophilic headgroups of the lipids, which induces swelling and sequential detachment of lipid layers, forming bilamellar vesicles with concentric bilayers.[1] Since the previous reports[2] shown the lipid membrane coated in the polyacrylamide gel facilitate to the uniformly and gently produce the floating giant unilamellar liposome, due to the help of the pore structure of the polyacrylamide gel film, the encapsulation of all components in loading-inner solution in liposome can be achieved. When using the neutral phospholipid DOPC to form liposomes, the encapsulation of inner reaction components was uniform, avoiding selective encapsulation based on charge differences.

Operation of AD-triggered transient reaction circuit in liposome assembly.

To follow the temporal fluorescent readout of reaction circuit in liposome, the 50 μL mixture of reaction circuit-loaded liposome was transferred into the cuvette and triggered by variable concentrations of AD (1 mM, 2 mM). The transient fluorescence kinetic of liposome solution was followed while mixing the testing liposome solution within 20-minute intervals.

Operation of AD-triggered transient DNAzyme circuit in liposome assembly.

Before the characterization of the operating DNAzyme circuits in liposome, the lipsome sample was irradiated undre UV light source (fixing the light intensity of 5 mW/cm^2) for 10 minutes to photo-cleaving the hairpin (H_{D1D2}) and generate the separated conpenents composed in AD-triggered DNAzyme. To follow the temporal fluorescent readout of DNAzyme circuit in liposome, the 50 μL mixture of DNAzyme circuit-loaded liposome was transferred into the cuvette and triggered by variable concentrations of AD (1 mM). The transient catalytic rate of fluorophore/quencher-modified substrate reporter of DNAzyme circuit in liposome was followed, with the testing liposome solution mixed at 15 minute-intervals.

Operation of AD-triggered transient evolution of CDN X in liposome assembly.

Before the characterization of the operating CDN X in liposome, the lipsome sample was irradiated undre UV light source (fixing the light intensity of 5 mW/cm^2) for 10

minutes to photo-cleaving the two hairpins ($H_{AA'}$, $H_{BB'}$) and generate the separated conpenents composed in AD-triggered CDN X. Then, the characterization of intra-liposome transient CDN X was followed by the fluorophore/quencher-modified substrate reporters associated with the corresponding constituents of CDN X. Taking FAM/BHQ1-modified Sub-1-based ($H_{AA'}$, $H_{BB'}$)-loaded liposome as an example, to follow the catalytic cleavage of Sub-1 by the constituent BA′ in transient evolution of CDN X in the liposome, the 50 μL mixture of the liposome solution was transferred into a cuvette and triggered with varying concentrations of AD (1 mM), with the testing liposome solution mixed at 15 minute-intervals.

Confocal characterization of AD-triggered transient reaction circuit, DNAzyme circuit, and evolution of CDN X in liposome assembly.

The intra-liposome transient systems were also followed by the Levi-Montalcini confocal microscope. Before the characterization of the operating DNAzyme circuits or CDN X in liposome, the lipsome sample was irradiated undre UV light source (fixing the light intensity of 5 mW/cm^2) for 10 minutes to photo-cleaving the hairpin (H_{D1D2}) or two hairpins ($H_{AA'}$, $H_{BB'}$) and generate the separated conpenents composed in AD-triggered DNAzyme circuits or CDN X. Aliquots of 3 μL were withdrawn from each 50 μL different systems-loaded liposome solutions upon triggered with varying concentrations of AD (1 mM). The corresponding confocal fluorescence images were captured at certain time-intervals.

Preparation of circuits I-IV and photoresponsive hairpin $H_{EE'}$-loaded liposomes modified with linking tether x.

A mixture of 2 micromoles of DOPC, 1 micromole of DOPE, and 1 micromole of cholesterol was dissolved in 2 mL of chloroform in a 5 mL glass vial. The chloroform was evaporated under a stream of nitrogen (N_2) to form a lipid film, which was then further dried under high vacuum. The lipid film was rehydrated using a reaction solution (4 mL, including circuits I-IV reaction solutions or photoresponsive hairpin $H_{EE'}$ reaction solution) and the suspension was vortexed. Taking circuit I reaction solution as example, this includes a mixture solution of 4 μM duplexes L_1'/F′ and L_2'/P, in 20 mM Tris-HCl Buffer (20 mM magnesium chloride, pH 7.38@25°C). The details of other circuits II-IV reaction solutions refer to Figure S22. The photoresponsive hairpins $H_{EE'}$ reaction solution includes 6 μM photoresponsive $H_{EE'}$ in 20 mM Tris-HCl Buffer (20 mM magnesium chloride, pH 7.38@25°C). To form a liposome suspension, the mixture was repeatedly extruded 20 times through a 220 nm polycarbonate membrane using a syringe. The resulting liposome suspension was diluted to form a liposome suspension with a final DOPC lipid concentration of 120 μM. Finally, the liposomes were incubated with cholesterol-modified nucleic acid (x) at 200 nM for 1 hour.

Cell experiments

Cell culture: Normal breast cells (MCF-10A) were maintained in complete growth medium consisting of 1:1 mixture of Dulbecco′s modified Eagle′s medium and Ham′s

F12 medium supplemented with horse serum (5%), epidermal growth factor (20 ng mL^{-1}), cholera toxin (CT, 0.1 μg mg^{-1}), insulin (10 μg mL^{-1}), hydrocortisone (500 ng mL^{-1}), and penicillin/streptomycin (1 unit mL^{-1}). Human breast cancer cells (MCF-7) were grown in 5% CO_2 RPMI-1640 medium supplemented with 10% FCS, L-glutamine, and antibiotics (Biological Industries). Cells were plated one day prior to the experiment on 96-well plates for cell viability.

Confocal Microscopy Measurements: For cell imaging experiments, cells were planted on a μ-slide 4-well glass bottom dish (ibidi). Cells were incubated with the circuits I-IV-loaded liposomes after being washed with phosphate-buffered saline. The three samples of circuits I-IV-loaded liposomes (4 μM) were incubated with cells for 5 h and then washed with DMEM/HEPES twice and replenished with the fresh medium for the measurement. The fluorescence of Cy3 (λ_{ex} = 550 nm; λ_{em} = 564 nm) in cells was monitored with an Olympus FV3000 confocal laser scanning microscope, and all images were analyzed with ImageJ.

Cell viability experiments: Cell viability was assayed after incubation of different loaded liposome systems (6 μM $H_{EE'}$) in MCF-10A, MCF-7 cells planted at a density of 1.2×10^4 cells per well in 96-well plates. Before the incubation, the MCF-10A cells and MCF-7 cells were treated with linker (200 nM), x-aptamer AS1411, to functionalize a linking tether x in the cell membrane. After incubation with the loaded liposomes for 4 hours, cells were washed intensively with growth medium. After washing, cells were replenished with full medium and further incubated for 24 hours. The cell viability was, then, determined with the fluorescent redox probe, Presto-Blue. The fluorescence of Presto-Blue was recorded on a plate-reader (Tecan Safire) after 1 h of incubation at 37 °C (λ_{ex} = 560 nm; λ_{em} = 590 nm).

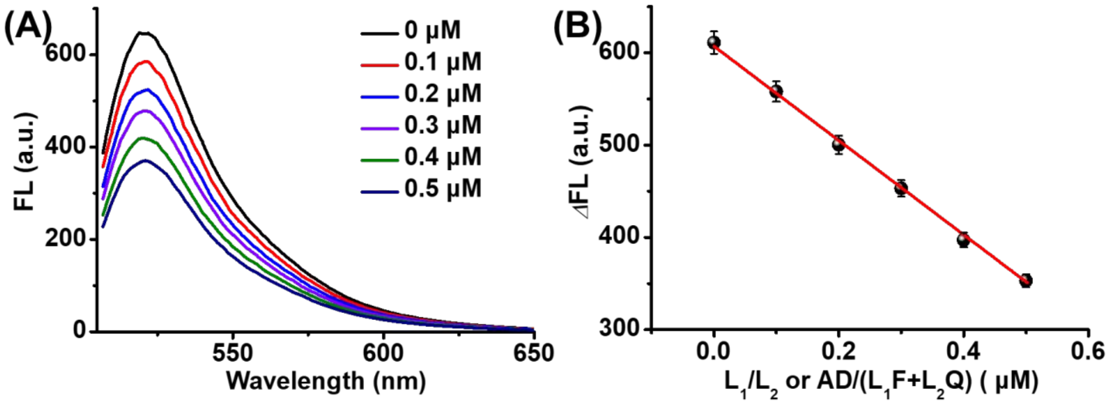

Figure S1. (A)The fluorescence spectra of AD/(L_1F+L_2Q) complex upon the addition of different concentrations of L_1 and L_2 in the presence of a mixture of 4 mM AD, 0.5 µM F and Q. (B) The derived calibration curve relating the fluorescence intensity of AD/(L_1F+L_2Q) complex to the different concentrations of L_1 and L_2. Error bars are derived from N = 3 independent experiments.

Note: The transient circuit system shown in Figure 1 was quantitatively evaluated using the calibration curve shown in Figure S1. The reaction solution in Figure 1 consisted of a mixture of 0.5 µM duplexes (L_1/F and L_2/Q) and 0.025 U/mL ADA in 20 mM Tris-HCl buffer (20 mM magnesium chloride, pH 7.38@25°C). The temporal fluorescence intensity of the intermediate AD/(L_1F+L_2Q) complex depended on the concentration of this AD-triggered complex in the presence of an initial 0.5 µM of disassembled duplexes (L_1/F and L_2/Q) and 4 mM AD. In other words, the fluorescence intensity changes reflect the increasing concentration of the assembled intermediate AD/(L_1F+L_2Q) complex and the decreasing concentration of separated duplexes (L_1/F and L_2/Q).

To construct the calibration curve, a reaction solution containing 0.5 µM F and Q as free-labeled strands and 4 mM AD in 20 mM Tris-HCl buffer (20 mM magnesium chloride, pH 7.38@25°C) was prepared. It should be noted that to derive the calibration curve shown in Figure S1B, the 4 mM AD was in excess relative to the 0.5 µM duplexes (L_1/F and L_2/Q) required for assembly. By adding varying concentrations of L_1 and L_2 to the calibration reaction mixture, the relationship between the known concentration of the AD/(L_1F+L_2Q) complex and fluorescence intensity was established, enabling the construction of a standard calibration curve to evaluate the transient concentration of the intermediate AD/(L_1F+L_2Q) complex in Figure 1.

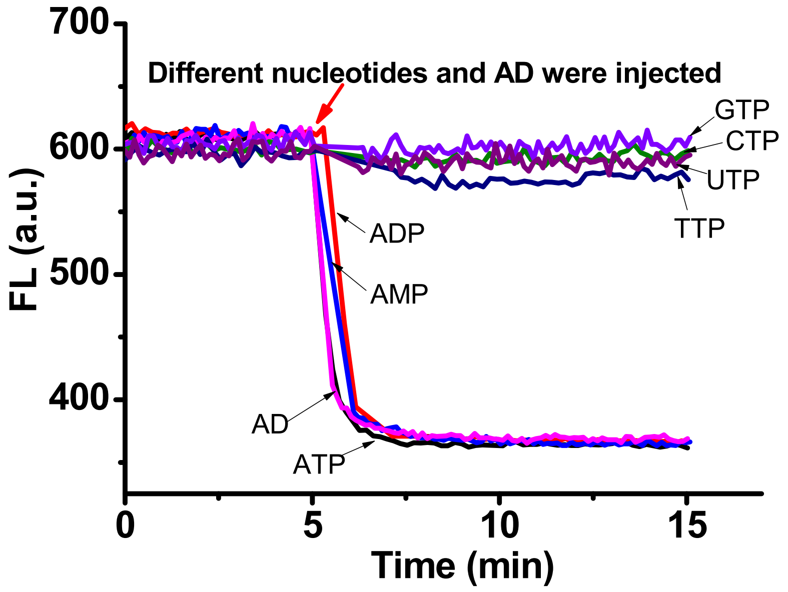

Figure S2. Time-dependent fluorescence intensity upon subjecting 4 mM different nucleotides including CTP, TTP, GTP, UTP and adenine-containing ligands, such as AMP, ADP or ATP to 0.5 μM (L_1F+L_2Q).

The evaluation of the selectivity of AD/AD-aptamer in transient circuits.

The binding affinity between AD and AD-aptamer is the main power to drive all the AD-triggered transient DNA circuits and CDX circuitry in the paper. The selectivity of the molecule/aptamer was evaluated by adding different nucleotides including CTP, TTP, GTP, UTP and adenine-containing ligands, such as AMP, ADP or ATP to 0.5 μM (L_1F+L_2Q) shown in Figure 1A. Figure S2 shows that the adenine-containing ligands (AMP, ADP or ATP) have the comparable affinities to the aptamer, leading to the decrease of fluorescence intensity by formation of the ligands/(L_1F+L_2Q) complex. On the contrary, the other nucleotides (CTP, TTP, GTP, and UTP) lacking any affinity to the aptamer does not result in fluorescence change of the system.

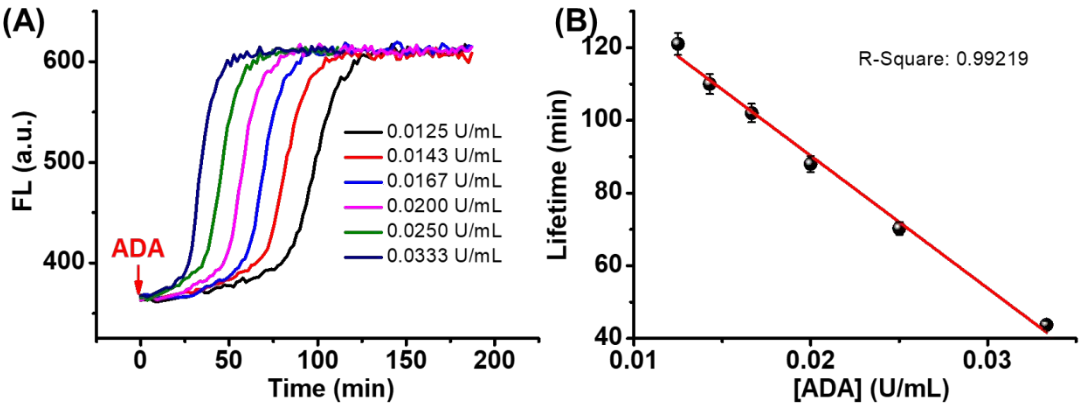

Figure S3. (A) Time-dependent fluorescence changes of the disassembly of AD (1.5 mM)/AD-aptamer subunits supramolecular complex upon subjecting different ADA concentrations: 0.0125, 0.0143, 0.0167, 0.0200, 0.0250, and 0.0333 U/mL. (B) The derived calibration curve relating the lifetime of disassembly of AD/(L_1F+L_2Q) complex to the different concentrations of ADA. Error bars are derived from N = 3 independent experiments.

The application of AD-triggered transient circuit for sensing ADA.

Since the ADA catalyzed AD into inosine lacking the affinity to the AD-aptamer, the disassembly of AD/(L_1F+L_2Q) complex result in resetting system into a beginning fluorescence intensity within a certain lifetime. Subjecting different concentrations of ADA to the AD-stabilized AD/(L_1F+L_2Q) complex solution, the variable “resetting” lifetimes were evaluated, Figure S3A. By relating the lifetime of disassembly of AD/(L_1F+L_2Q) complex to the different concentrations of ADA, an AD-related calibration curve was derived, Figure S3B. Therefore, the sensing platform demonstrates capability in detecting ADA with a low detection limit of 0.0125 U/mL. This sensitivity level is either superior to or on par with other detection methods reported in the literature. The sensing platform in ADA detection showcases its potential as a valuable tool for various analytical and diagnostic applications.

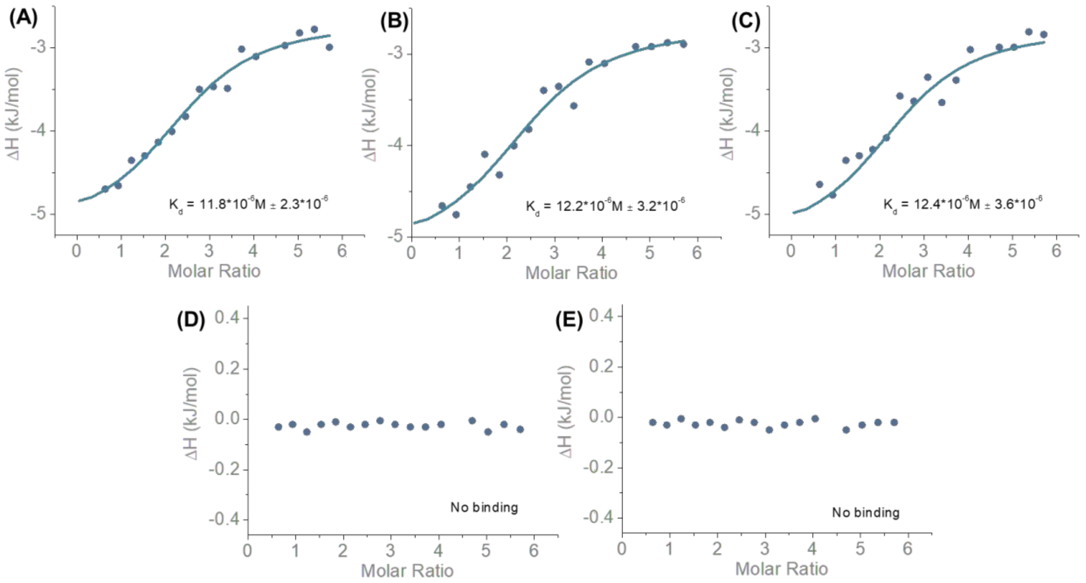

Figure S4. Isothermal titration calorimetry (ITC) plots of molar enthalpy change vs molar ratio of: (A) (L_1+L_2) and AD; (B) (L_1+L_2) and AD in presence of denatured ADA; (C) (L_1+L_2) and AD in presence of inosine; (D) (L_1+L_2) and ADA; (E) (L_1+L_2) and inosine.

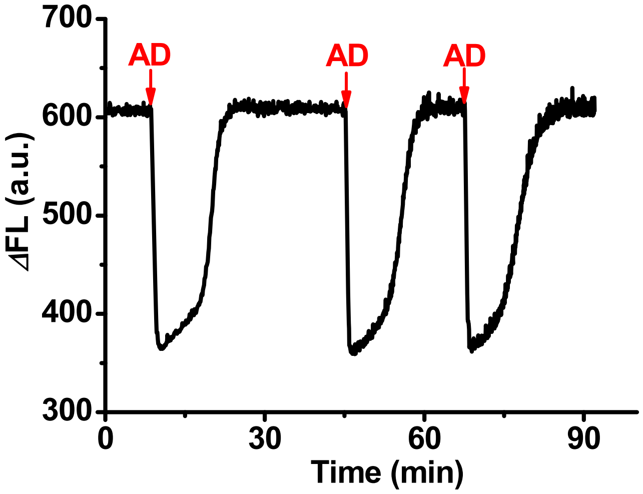

Figure S5. Time-dependent fluorescence changes of AD/(L_1F+L_2Q) supramolecular complex in presence of 0.2 U/mL ADA upon cyclic treatment of 2 mM AD.

Kinetic equations of AD-triggered transient reaction circuit shown in Figure 1A. The kinetic scheme of reactions associated with the time-dependent concentration changes of AD/(L_1F+L_2Q) intermediate in AD-triggered reaction circuit is summarized in the following equations. Knowing the time-dependent concentration changes of each constituent, we computationally simulated the time-dependent concentration changes by using Matlab R2019b.

$$(1) L_1F + L_2Q + AD \underset{k_{-1}}{\overset{k_1}{\rightleftarrows}} L_1F.L_2Q.AD$$

$$(2) AD + ADA \underset{k_{-2}}{\overset{k_2}{\rightleftarrows}} AD.ADA$$

$$(3) AD.ADA \xrightarrow{k_3} \text{Inosine} + ADA$$

Derivatives:

$$\frac{dL_1F}{dt} = K_{-1}[L_1F.L_2Q.AD] - K_1[L_1F][L_2Q][AD]$$

$$\frac{dL_2Q}{dt} = K_{-1}[L_1F.L_2Q.AD] - K_1[L_1F][L_2Q][AD]$$

$$\frac{dAD}{dt} = K_{-1}[L_1F.L_2Q.AD] - K_1[L_1F][L_2Q][AD] + K_{-2}[AD.ADA] - K_2[AD][ADA]$$

$$\frac{dL_1F.L_2Q.AD}{dt} = K_1[L_1F][L_2Q][AD] - K_{-1}[L_1F.L_2Q.AD]$$

$$\frac{dADA}{dt} = K_{-2}[AD.ADA] - K_2[AD][ADA] + K_3[AD.ADA]$$

$$\frac{dAD.ADA}{dt} = K_2[AD][ADA] - K_{-2}[AD.ADA] - K_3[AD.ADA]$$

$$\frac{d\text{Inosine}}{dt} = K_3[AD.ADA]$$

Figure S6. The kinetic scheme of the reactions associated with the time-dependent concentration changes of AD/(L_1F+L_2Q) is summarized in above equations. Knowing the time-dependent concentration changes of the complex AD/(L_1F+L_2Q), we computationally simulated the time-dependent concentration changes by using Matlab R2019b.

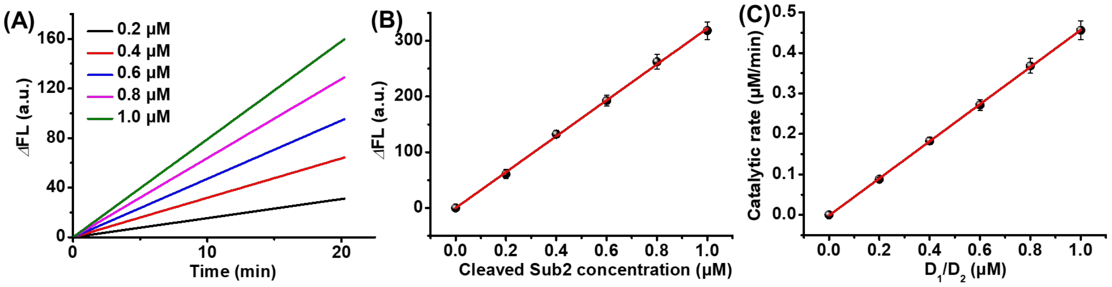

Figure S7. (A) Time-dependent fluorescence changes generated from the cleavage of the fluorophore (F_i)/quencher (Q_i)-modified substrates by the DNAzyme unit associated with assembled AD/(D_1+D_2) complex at variable concentrations of D_1 and D_2 in presence of 4 mM AD. (B) Calibration curve of background-subtracted fluorescence intensity versus cleaved-sub2 concentration. (C) Calibration curves of AD/(D_1+D_2) complex as a function of their concentrations. Error bars are derived from N = 3 independent experiments.

Kinetic equations of AD-triggered transient DNAzyme circuit shown in Figure 2A. The kinetic scheme of reactions associated with the time-dependent concentration changes of AD/(D_1+D_2) intermediate DNAzyme is summarized in the following equations. Knowing the time-dependent concentration changes of each constituent, we computationally simulated the time-dependent concentration changes by using Matlab R2019b.

$$(1) D_1 + D_2 + AD \underset{k_{-1}}{\overset{k_1}{\rightleftarrows}} D_1D_2AD$$

$$(2) AD + ADA \underset{k_{-2}}{\overset{k_2}{\rightleftarrows}} AD.ADA$$

$$(3) AD.ADA \xrightarrow{k3} \text{Inosine} + ADA$$

Derivatives:

$$\frac{dD_1}{dt} = K_{-1}[D_1D_2AD] - K_1[D_1][D_2][AD]$$

$$\frac{dD_2}{dt} = K_{-1}[D_1D_2AD] - K_1[D_1][D_2][AD]$$

$$\frac{dAD}{dt} = K_{-1}[D_1D_2AD] - K_1[D_1][D_2][AD] + K_{-2}[AD.ADA] - K_2[AD][ADA]$$

$$\frac{dD_1D_2AD}{dt} = K_1[D_1][D_2][AD] - K_{-1}[D_1D_2AD]$$

$$\frac{dADA}{dt} = K_{-2}[AD.ADA] - K_2[AD][ADA] + K_3[AD.ADA]$$

$$\frac{dAD.ADA}{dt} = K_2[AD][ADA] - K_{-2}[AD.ADA] - K_3[AD.ADA]$$

$$\frac{d\text{Inosine}}{dt} = K_3[AD.ADA]$$

Figure S8. The kinetic scheme of the reactions associated with the time-dependent concentration changes of AD/(D_1+D_2) is summarized in above equations. Knowing the time-dependent concentration changes of the AD/(D_1+D_2) DNAzyme intermediate, we computationally simulated the time-dependent concentration changes by using Matlab R2019b.

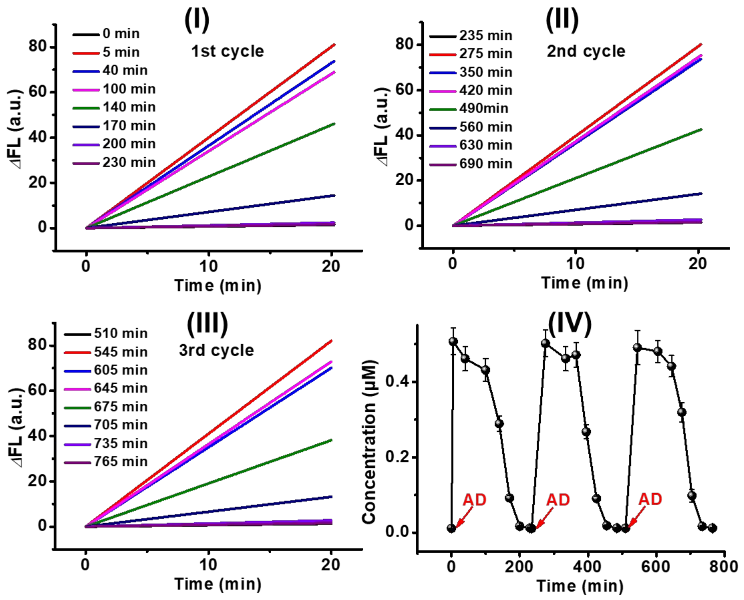

Figure S9. Time-dependent fluorescence intensity changes generated by the DNAzyme-catalyzed cleavage of the F_i/Q_i-modified substrate by samples withdrawn from the reaction mixture of operating the transient catalytic circuit at time-intervals. Panel I-Reaction circuit activated by AD, 3 mM. Panel II-Reaction circuit re-activated by AD, 3 mM at time interval 230 min. Panel III-Reaction circuit re-activated by AD, 3 mM at time interval 505 min. Panel IV-Transient concentration changes of the AD-stabilized Mg^{2+}-ion-DNAzyme in double injection of 3 mM AD.

The evaluation of the stability of AD-triggered transient DNAzyme circuit.

The AD-triggered transient DNAzyme circuit exhibits sustained dynamic behavior through multiple cycles of fuel (AD) addition. As shown in Figure S9, Panels I–III, the DNAzyme-catalyzed cleavage of the F_i/Q_i-modified substrate is maintained over time, with fluorescence intensity changes observed upon initial activation with AD (Panel I) and subsequent re-activations at 230 min (Panel II) and 505 min (Panel III). Furthermore, Panel IV illustrates the transient concentration changes of the AD-stabilized Mg^{2+}-ion-DNAzyme upon operation of 3 mM AD-triggered cycles, demonstrating that the circuit can be re-fueled and retain its catalytic functions for at least three cycles.

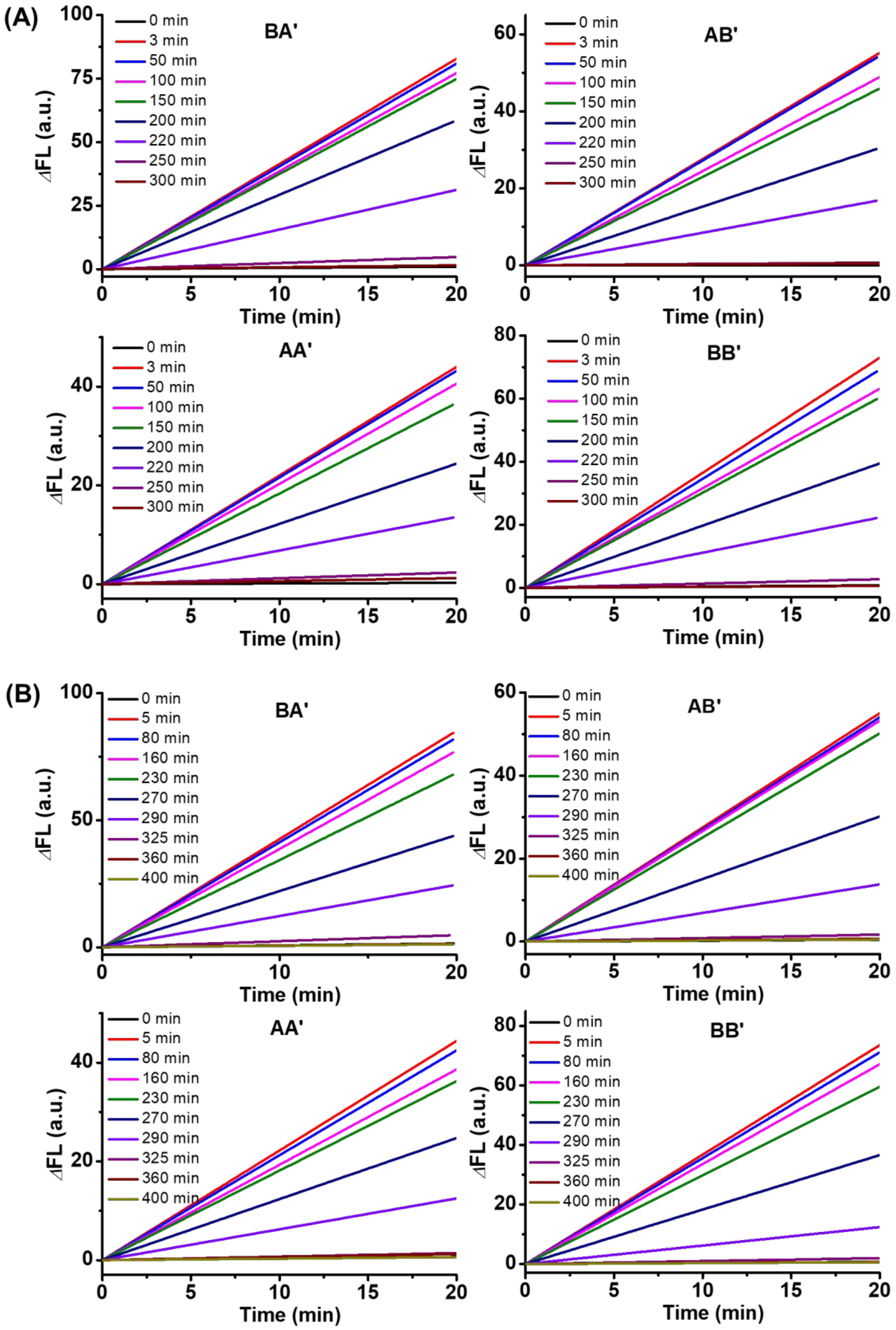

Figure S10. Time-dependent fluorescence changes of Q_x/F_x-modified substrate cleaved by the DNAzyme reporter units at different time intervals of transient AD-triggered transient evolution of CDN X in presence of: (A) 4 mM AD, (B) 5 mM AD.

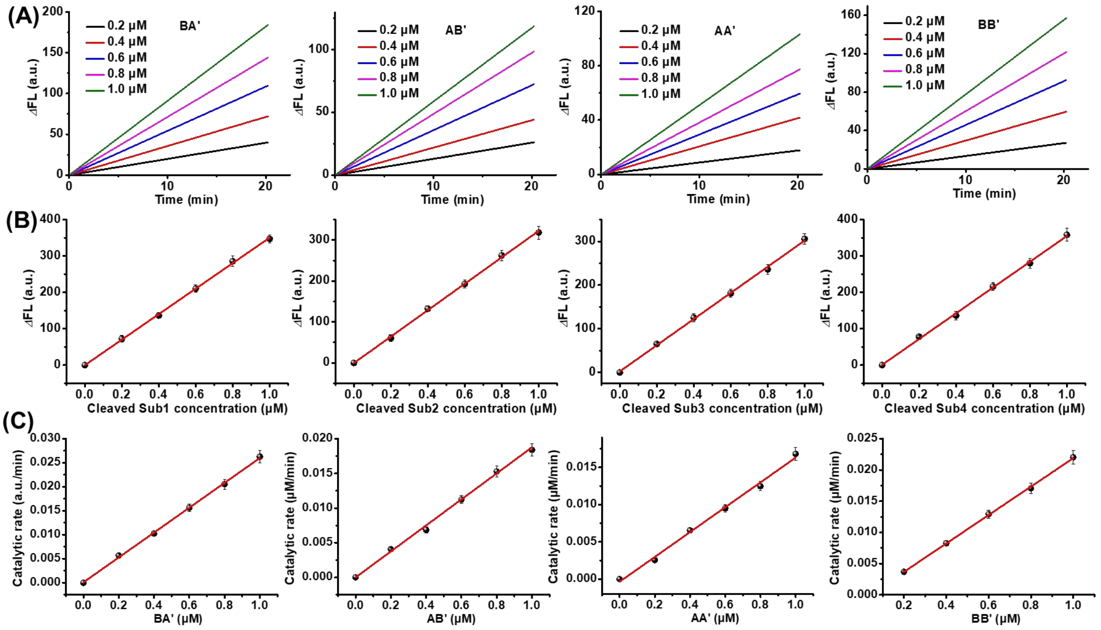

Figure S11. (A) Time-dependent fluorescence changes generated from the cleavage of the fluorophore (F_x)/quencher (Q_x)-modified substrates by the respective DNAzyme reporter units associated with the individual constituents (of CDN X) at variable concentrations. (B) Calibration curves of background-subtracted fluorescence intensity versus cleaved-substrate concentrations. (C) Calibration curves of constituents in CDN X as a function of their concentrations. Error bars are derived from N = 3 independent experiments.

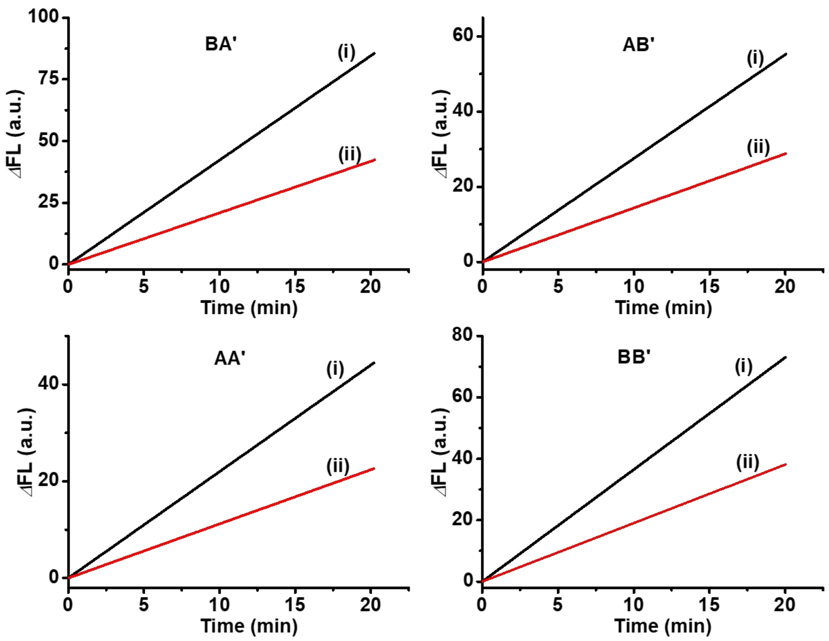

Figure S12. Time-dependent fluorescence changes of Q_x/F_x-modified substrate cleaved by the DNAzyme reporter units of AD-triggered evolution of CDN X in presence of 4 mM AD under (i) 20 mM Mg^{2+} and (ii) 5 mM Mg^{2+}.

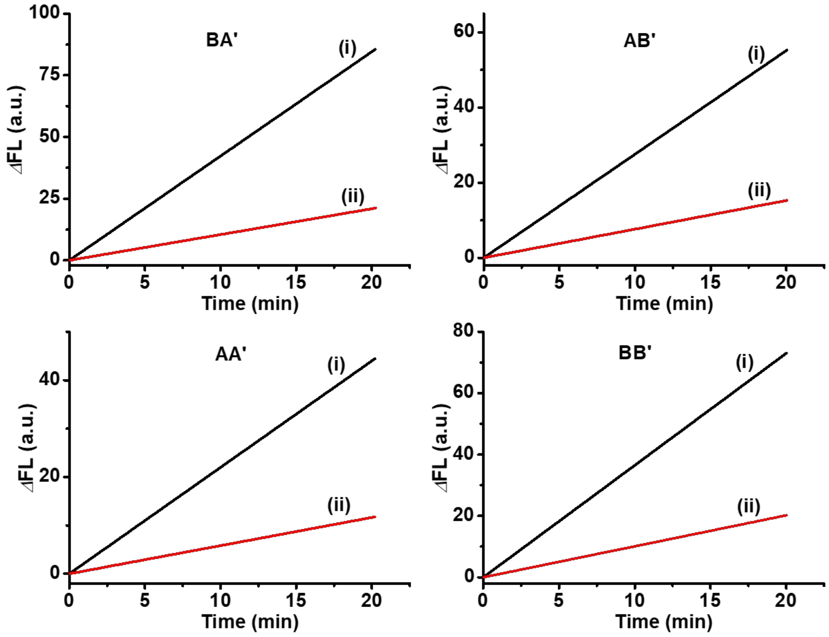

Figure S13. Time-dependent fluorescence changes of Q_x/F_x-modified substrate cleaved by the DNAzyme reporter units of AD-triggered evolution of CDN X in presence of 4 mM AD under (i) 25 ℃ and (ii) 30 ℃.

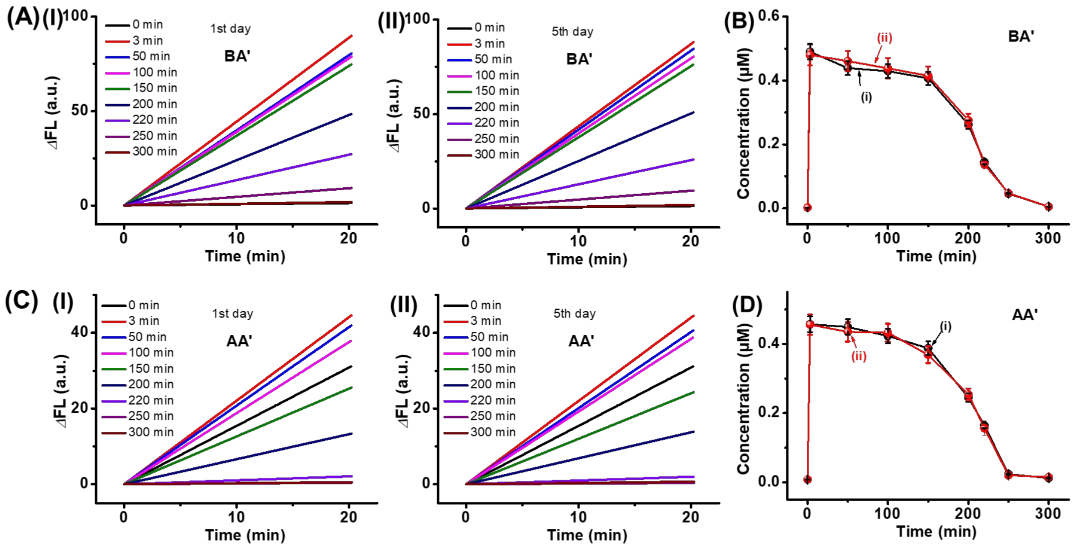

Figure S14. (A) Time-dependent fluorescence changes of the Q_x/F_x-modified substrate cleaved by BA′ constituents during the AD-triggered transient evolution of CDN X in the presence of 4 mM AD and 0.025 U/mL ADA. Panel I: Experiment performed on day 1; Panel II: Experiment performed on day 5. (B) Transient concentration changes of constituent BA′ in CDN X measured in samples collected at specified time intervals. Curve (i): Experiment performed on day 1; Curve (ii): Experiment performed on day 5. (B) Time-dependent fluorescence changes of the Q_x/F_x-modified substrate cleaved by AA′ constituents during the AD-triggered transient evolution of CDN X in the presence of 4 mM AD and 0.025 U/mL ADA. Panel I: Experiment performed on day 1; Panel II: Experiment performed on day 5. (B) Transient concentration changes of constituent AA′ in CDN X measured in samples collected at specified time intervals. Curve (i): Experiment performed on day 1; Curve (ii): Experiment performed on day 5. Error bars are derived from N = 3 independent experiments.

The evaluation of stability and functionality of the CDN X reaction system.

To evaluate the stability of the AD/ADA-driven CDN X circuit, we prepared two pieces of large volume of “Rest” reaction solution including 1 μM each of the hairpin strands A, A′, B, B′, and 0.025 U/mL ADA enzyme in a buffer comprising 20 mM Tris-HCl, 20 mM magnesium chloride. The two pieces of the reaction solution were separately triggered by 4 mM AD on first day and fifth day, respectively. According to the above measuring procedure of CDN X system, the transient dynamic concentration changes of two constituents (BA′ and AB′) of CDN X were followed to evaluate the stability and functional behavior of the transient CDN X circuit. Figure S14A, panels I and II, present the cleavage rates of the F_x/Q_x-modified substrates by the DNAzyme “reporter” units of constituent BA′ on the first and fifth days, respectively. Similarly, Figure S14C, panels I and II, present the cleavage rates of the F_x/Q_x-modified substrates by the DNAzyme “reporter” units of constituent AA′ on the first and fifth days, respectively. These rates were measured in samples taken at intervals during the transient dynamic evolution of the CDN X reaction triggered by AD. By applying the corresponding calibration curve shown in Figure S11, the dynamic concentration changes of the constituents (BA′, AA′) on first day and fifth day are derived shown in Figures S14B

and S14D, respectively. Ignorable differences of the dynamic behavior of transient CDN X circuits are found upon activating the CDN X circuits after long-term storage, demonstrating the good stability of the components within the CDN X system and its function.

Kinetic equations of AD-triggered transient CDN X shown in Figure 3A.

The kinetic scheme of reactions associated with the time-dependent concentration changes of four constituents associated with the AD-triggered transient evolution of CDN X is summarized in the following equations. Knowing the time-dependent concentration changes of each constituent, we computationally simulated the time-dependent concentration changes by using Matlab R2019b.

$$(1) A + A' + AD \underset{k_{-1}}{\overset{k_1}{\rightleftarrows}} AA'AD$$

$$(2) B + B' + AD \underset{k_{-2}}{\overset{k_2}{\rightleftarrows}} BB'AD$$

$$(3) A + B' + AD \underset{k_{-3}}{\overset{k_3}{\rightleftarrows}} AB'AD$$

$$(4) B + A' + AD \underset{k_{-4}}{\overset{k_4}{\rightleftarrows}} BA'AD$$

$$(5) AA'AD + BB'AD \underset{k_{-5}}{\overset{k_5}{\rightleftarrows}} AB'AD + BA'AD$$

$$(6) AD + ADA \underset{k_{-6}}{\overset{k_6}{\rightleftarrows}} AD.ADA$$

$$(7) AD.ADA \xrightarrow{k_7} \text{Inosine} + ADA$$

Derivatives:

$$\frac{dA}{dt} = K_{-1}[AA'AD] - K_1[A][A'][AD] + K_{-3}[AB'AD] - K_3[A][B'][AD]$$

$$\frac{dA'}{dt} = K_{-1}[AA'AD] - K_1[A][A'][AD] + K_{-4}[BA'AD] - K_4[B][A'][AD]$$

$$\frac{dAD}{dt} = K_{-1}[AA'AD] - K_1[A][A'][AD] + K_{-2}[BB'AD] - K_2[B][B'][AD] + K_{-3}[AB'AD]$$
$$-K_3[A][B'][AD] + K_{-4}[BA'AD] - K_4[B][A'][AD] + K_{-6}[AD.ADA] - K_6[AD][ADA]$$

$$\frac{dAA'AD}{dt} = K_1[A][A'][AD] - K_{-1}[AA'AD] + K_{-5}[AB'AD][BA'AD] - K_5[AA'AD][BB'AD]$$

$$\frac{dB}{dt} = K_{-2}[BB'AD] - K_2[B][B'][AD] + K_{-4}[BA'AD] - K_4[B][A'][AD]$$

$$\frac{dB'}{dt} = K_{-2}[BB'AD] - K_2[B][B'][AD] + K_{-3}[AB'AD] - K_3[A][B'][AD]$$

$$\frac{dBB'AD}{dt} = K_2[B][B'][AD] - K_{-2}[BB'AD] + K_{-5}[AB'AD][BA'AD] - K_5[AA'AD][BB'AD]$$

$$\frac{dAB'AD}{dt} = K_3[A][B'][AD] - K_{-3}[AB'AD] + K_5[AA'AD][BB'AD] - K_{-5}[AB'AD][BA'AD]$$

$$\frac{dBA'AD}{dt} = K_4[B][A'][AD] - K_{-4}[BA'AD] + K_5[AA'AD][BB'AD] - K_{-5}[AB'AD][BA'AD]$$

$$\frac{dADA}{dt} = K_{-6}[AD.ADA] - K_6[AD][ADA] + K_7[AD.ADA]$$

$$\frac{dAD.ADA}{dt} = K_6[AD][ADA] - K_{-6}[AD.ADA] - K_7[AD.ADA]$$

$$\frac{d\text{Inosine}}{dt} = K_7[AD.ADA]$$

Figure S15. The kinetic scheme of the reactions associated with the time-dependent concentration changes of AD-triggered transient evolution of CDN X is summarized in above equations. Knowing the time-dependent concentration changes of each constituent, we computationally simulated the time-dependent concentration changes by using Matlab R2019b.

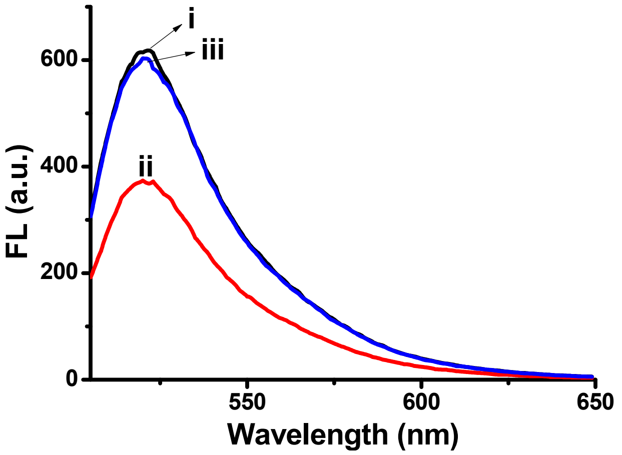

Figure S16. The spectra of the fluorescent mixtures of L_1F (0.5 μM) and L_2Q (0.5 μM) in presence of: (i) 0 mM AD, (ii) 4 mM AD, and (iii) 4 mM AD and 1 μM DNA inhibitor.

The function of DNA strand as inhibitor in regulation of AD-triggered transient circuit.

A promising approach for developing potential inhibitors that target the specific AD-aptamer interaction could involve utilizing DNA strands that are complementary to key aptamer domains. This complementary strand would be designed to interfere with the binding of the AD with (L_1F+ L_2Q), thereby disrupting the activation of the circuits. As we can see from the Figure S16, The fluorescent intensity of the module solution remains negligible in the presence of 1 μM DNA inhibitors, demonstrating the efficient prevention of duplexes L_1F and L_2Q from forming the AD/(L_1F + L_2Q) complex.

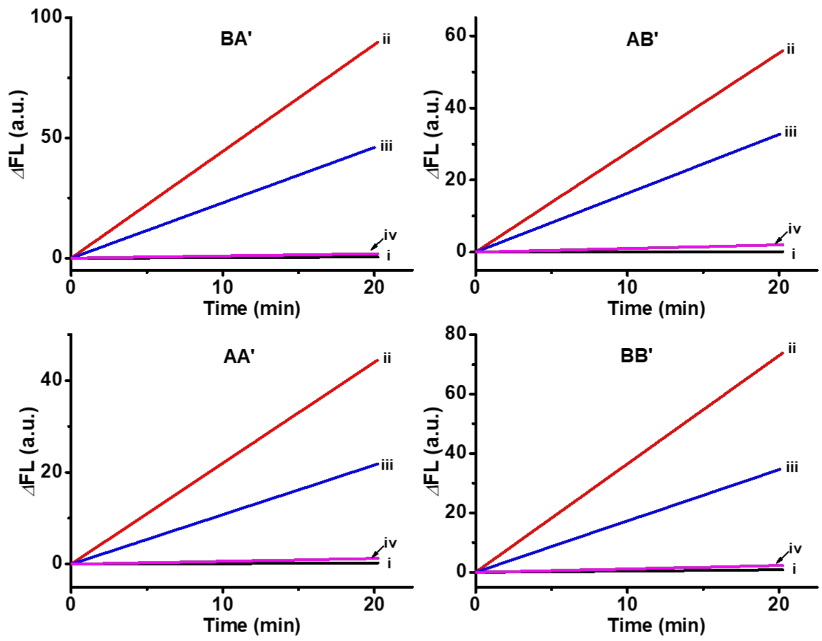

Figure S17. Time-dependent fluorescence changes of Q_x/F_x-modified substrate cleaved by the DNAzyme reporter units of AD-triggered evolution of CDN X in presence of: (i) 0 mM AD, (ii) 4 mM AD, (iii) 4 mM AD and 1 µM DNA inhibitor, and (iv) 4 mM AD and 2 µM DNA inhibitor.

The function of DNA strand as inhibitor in regulating function of AD-triggered transient CDN X circuit.

The introduction of complementary DNA strands interferes with the binding between AD and its aptamer domains in components A, A′, B, and B′. This interference consequently disrupts the activation of the catalytic activities of CDN X circuits. As illustrated in Figure S17, the catalytic rates of the individual constituents of CDN X (namely BA′, AB′, AA′, and BB′) exhibit a progressive inhibition as the concentration of the DNA inhibitor increases. This observation demonstrates the effectiveness of the complementary DNA strands in modulating the aptamer-based dynamic circuits.

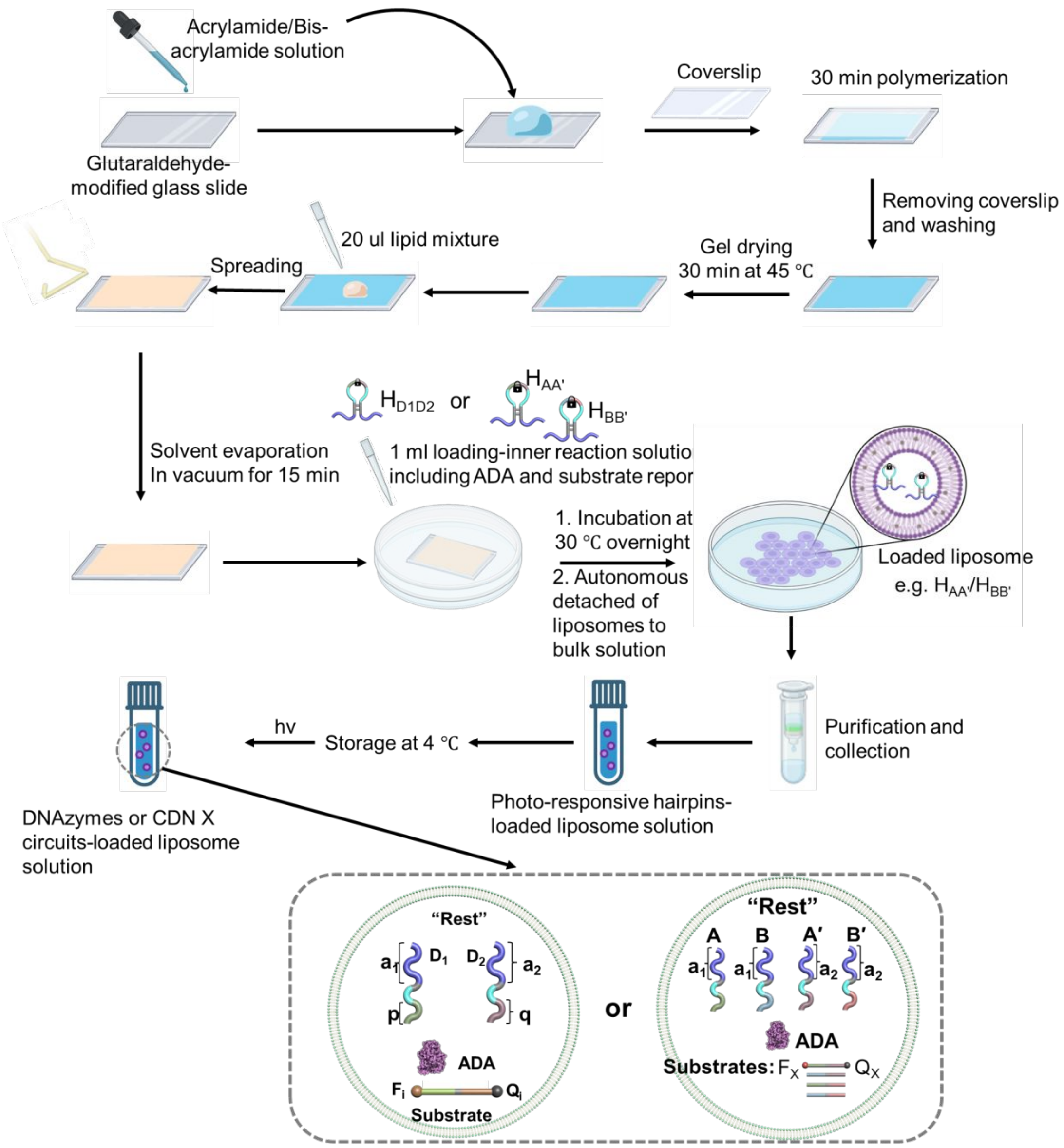

Figure S18. Procedure of DNAzyme circuits- and CDN X-loaded liposomes using dried polyacrylamide gels.

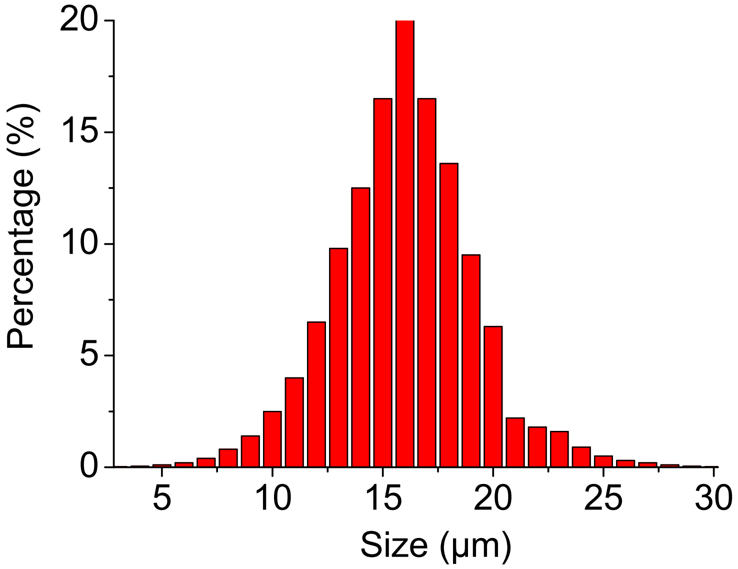

Figure S19. The size distribution of prepared liposome containments with a reaction module consisting of L_1F, L_2Q duplexes and ADA.

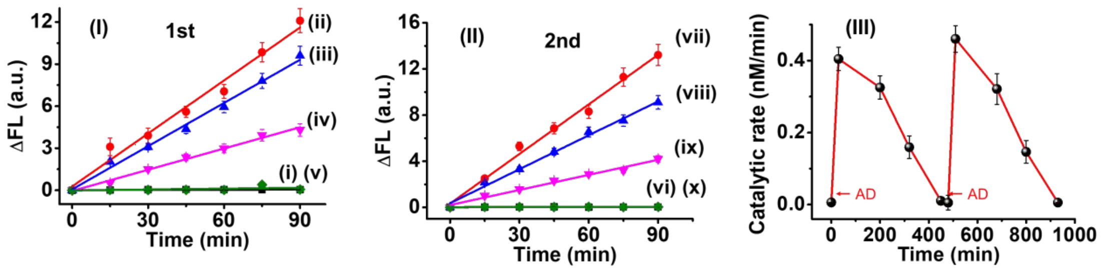

Figure S20. Panel I-Time-dependent fluorescence change generated at time-intervals by the transiently operating DNAzyme reaction module integrated in the liposome assembly at first AD-fueled cycle. (i-v) t = 0, 30, 200, 300, and 450 minutes. Panel II-Time-dependent fluorescence change generated at time-intervals by the transiently operating DNAzyme reaction module integrated in the liposome assembly at second AD-fueled cycle. (vi-x) t = 0, 30, 200, 300, and 450 minutes. The fluorescence of the liposomes is auto-zero prior to each time-interval of measurement: (i-v) t = 480, 510, 680, 800, and 930 minutes. Panel III-Cyclic transient catalytic rates corresponding to the cleavage of the F_i/Q_i-substrate at time-intervals of the DNAzyme operation in liposomes. Error bars are derived from three independent experiments.

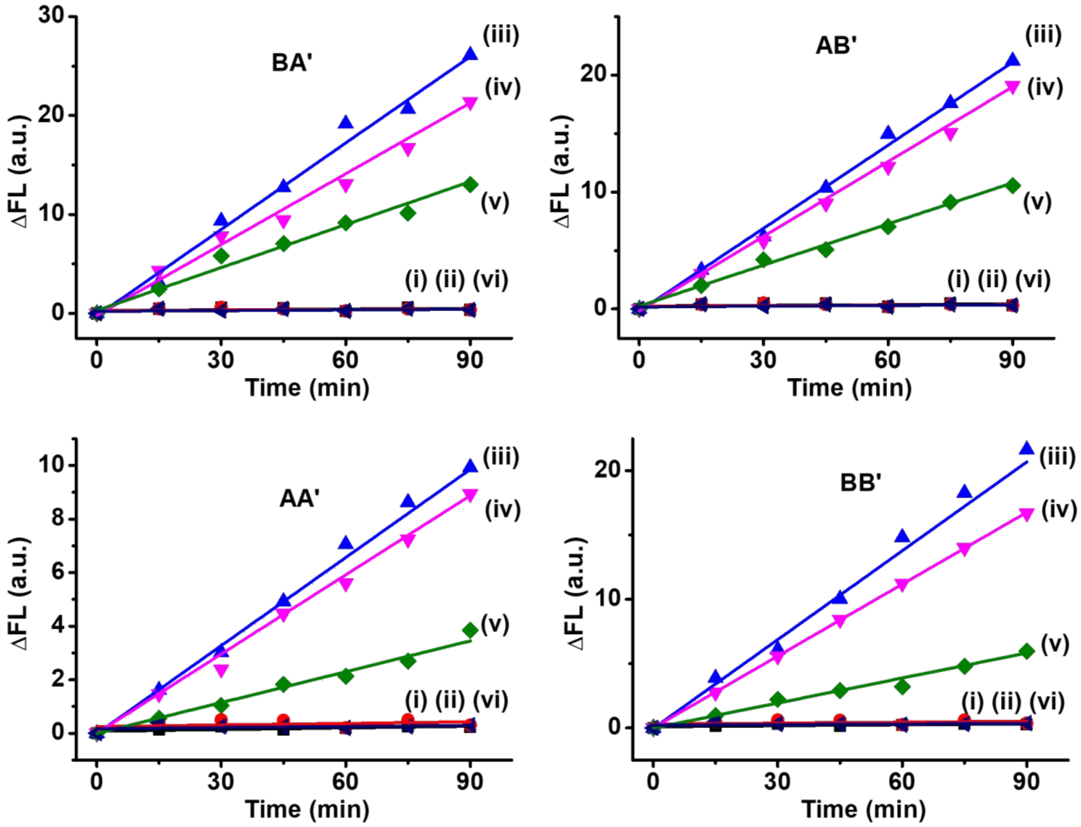

Figure S21. Time-dependent fluorescence change generated at time-intervals by constituents associated with transiently operating AD-triggered CDN X reaction module integrated in the liposome assembly. The fluorescence of the respective liposomes is auto-zero prior to each time-interval of measurement: (ii-vi) t = 0, 30, 200, 300, and 450 minutes.

Development of liposomes loaded with functional allosteric ATP-stabilized AD-aptamer subunits DNAzymes for selective gene therapy (Light-stimulated evolution of the DNAzyme cleaving EGR-1 mRNA leading to cancer cells apoptosis)

The systems discussed in the paper demonstrated AD-triggered allosteric activation of catalytic DNA circuits and of constitutional dynamic networks, and the capacity to evolve these circuits by light-triggered deprotection of photoresponsive caged hairpin structures. Moreover, we highlighted the light-induced integration of these circuits and networks into liposome assemblies. Furthermore, besides the selectivity of AD to trigger the frameworks, our studies revealed that adenine-containing agents, such as ATP, ADP and AMP are recognized by the AD aptamer subunits and can substitute adenosine. That is, ATP has the potential to act like the AD ligand that allosterically stabilizes the DNAzyme circuit or the constituents of the constitutional dynamic network. With this knowledge, we made an effort to adopt the features of the systems described in the study to design a framework reflecting potential medical applications related to cancer therapy. The system is schematically displayed in Figure 8. (1,2-di-(9Z-octadecenoyl)-sn-glycero-3-phosphocholine (DOPC)/1,2-dioleoyl-sn-glycero3-phosphoethanolamine (DOPE) liposomes are loaded with the photoresponsive caged hairpins $H_{EE'}$ and the boundaries of the liposomes are functionalized with cholesterol-modified nucleic acid tethers x. The MCF-7 breast cancer cells or MCF-10A epithelial breast cells are employed as uptake cancer cells or control cells for treatment. The cells are treated with the AS11411 aptamer conjugated to the strand x′, complementary to x. As the MCF-7 cells include in their membrane boundary the nucleolin receptor, recognized by the AS1411 aptamer, the strand x-modified liposomes hybridize with the x′-AS1411 aptamer units-functionalized MCF-7 cancer cells, resulting in the fusion of the liposomes with the cells, and the transfer of their loads into the cell cytoplasm. Note, however, that the fusion process of the liposomes, and the accompanying delivery of the loads into the MCF-10A epithelial cells is anticipated to inefficient due to the lack of membrane-associated nucleolin receptors in these cells. The $H_{EE'}$-loaded cells are, then, photo-deprotected to yield the separated the strands E and E′. The hairpin $H_{EE'}$ is, however, pre-engineered to include in the separated strands E, E′ comprising the AD (or ATP) aptamer subunits a_1/a_2, conjugated to the Mg^{2+}-ion-DNAzyme units linked to tethers b_1/b_2 as recognition sequences of the EGR-1 mRNA. Accordingly, ATP, overexpressed in the cancer cells, assembles the ATP-triggered allosteric activation of the DNAzyme cleaving the EGR-1 mRNA in the cytoplasm. Cleavage of the mRNA is, then, anticipated to lead to apoptosis of the cancer cells. The selectivity of cancer cell apoptosis originates from the selective aptamer-guided fusion of the functional liposomes into the cancer cells. The development of the proposed ATP-triggered allosteric activation of cleavage of EGR-1 mRNA involves two steps: (i) Confirming the selective x/x′-duplex induced fusion of liposomes with the MCF-7 cells and the delivery of loads. (ii) Demonstrating the selective ATP-guided allosteric cleavage of EGR-1 mRNA and accompanying MCF-7 cell apoptosis. Accordingly, the following experiments using the MCF-7/MCF-10A cells and appropriate control systems were

performed to follow the fusion process and the subsequent resulting apoptosis of the respective cells.

In the first step, the fusion of the x-tethered liposomes with the x′-tethered MCF-7 cancer cells and the MCF-10A epithelial normal cells, and the efficacy of transferring loads into the respective cells were examined. Towards these goals, liposomes loaded with the fluorescent modules shown in Figure S22A were prepared. The loads included the fluorescent constituents L_1'/F' and L_2'/P (circuits I and II), where a_1, a_2 are the AD aptamer subunits and strand F′ is modified with fluorophore Cy3. Alternatively, reaction modules L_1'/F' and L_2'/Q were loaded in control liposome (circuits III and IV), where strand Q is modified with quencher BHQ1. The liposomes loaded with circuits I and III lacked in their membrane the strands x as modifier, whereas the liposomes loaded with circuits II and IV were modified at their boundaries with the tethers x. The MCF-7 cells and MCF-10A epithelial cells were treated with the x′-AS1411 aptamer conjugate and then subjected to the liposomes loaded with the respective circuits I-IV, to induce potential fusion and respective load delivery. Note that, upon delivery of the loads into the cancer cells containing overexpressed ATP, the resulting ATP/(a_1+a_2) aptamer supramolecular complex is anticipated to yield a fluorescent complex of circuits I and II if delivered into the cells, whereas the delivered control circuits III and IV will exist in a non-fluorescent, quenched configuration. The results of fusion and delivery of the circuits loaded into the respective cell lines are presented in Figure S22B. In panel I, the fusion/delivery of the circuits loaded into the MCF-7 cells are presented. The fusion/delivery of the circuit I-loaded liposomes results in weak intracellular fluorescence, whereas the fusion/delivery of the circuit II-loaded liposomes demonstrates high intracellular fluorescence, consistent with the enhanced x/x′-fusion and load delivery of the liposomes with the MCF-7 cells. In the contrast, the MCF-7 cells treated with the control liposomes loaded with circuits III and IV show low fluorescence, consistent with the fluorescence-quenched configuration of ATP/$L_1'F'$+$L_2'Q$ supramolecular complex. Figure S22B, panel II shows the fluorescence of the MCF-10A epithelial cells treated with the liposomes loaded with circuits I-IV. All systems reveal negligible fluorescence, indicating the lack of fusion/delivery of the loads into these cells. These results are consistent with the fact that the epithelial MCF-10A cells lack nucleolin receptors to facilitate the fusion and loads delivery. Figure S23 summarizes in a column presentation the fluorescence intensities associated with the fusion and load delivery features of the different liposomes loaded with the respective fluorescent modules. The following important conclusions are derived from these experiments: (i) The modification of the liposomes with the x-tether and of the cells with the x′-AS1411 aptamer conjugate, significantly improves the fusion of liposomes with cells and load delivery process. (ii) The ATP overexpressed in cancer cells allows the intracellular ATP-driven formation of the AD-(ATP) aptamer subunits supramolecular complex.

The effective and selective fusion of the liposomes with MCF-7 cancer cells, by means of the x/x′-AS1411 aptamer duplex, suggested that by appropriate engineering of the load in the liposome containment with a ATP-responsive DNAzyme cleaving

mRNA, the liposome-delivered DNAzyme could intervene with cell functions. Specifically, one could design a DNAzyme cleaving the EGR-1 mRNA, leading to the dictated apoptosis of the cancer cells. Note that, the selective fusion of the liposomes dictated by the x/x′-AS1411 aptamer duplex could, then, provide a selective path for gene therapy.

Accordingly, liposomes were loaded with the photoresponsive o-nitrobenzyl phosphate-ester caged hairpins $H_{EE'}$ that include interlocked "caged" inactive strands E and E′ comprising the AD-aptamer subunits, a_1 and a_2, and DNAzyme binding-arm domains, b_1 and b_2, Figure 8. The liposomes are functionalized with the cholesterol-modified strand x. Breast cancer MCF-7 cells or epithelial breast cells MCF-10A were modified with the X′-AS1411 aptamer units and subjected to fusion with two alternative configurations of hairpin-loaded liposomes. One configuration (configuration I) included pre-irradiated liposomes (λ = 365 nm) in which the hairpin units were separated into strand E, E′ prior to fusion and delivery into the cells, Figure 9A, Panel I. In a second configuration (configuration II), the caged hairpin-loaded liposomes were fused with the cell contaminants, resulting in the delivery of the caged hairpins $H_{EE'}$ into the cell containments. The subsequent irradiation (λ = 365 nm) of the delivered $H_{EE'}$ hairpin structures in the cell containments yields, then, the separated strand E and E′. Note that, either fusion of the pre-irradiated liposomes (configuration I) or the fusion of the photoresponsive caged $H_{EE'}$ hairpins-loaded liposomes into the cells, followed by irradiation (configuration II) yield cellular containments containing separated strands E and E′. In the presence of overexpressed ATP in the MCF-7 cancer cells, the intracellular allosteric ATP-stabilized Mg^{2+}-ion-DNAzyme structure cleaving the EGR-1 mRNA is formed. Cleavage of the EGR-1 mRNA leads, then, to the apoptosis of the MCF-7 cells. Note that the lack of nucleolin receptors in the MCF-10A epithelial cells is anticipated to prohibit the fusion of the two different configurations of $H_{EE'}$-loaded liposomes, thus eliminating the cleavage of the mRNA in the epithelial cells. That is, the lack of fusion of the liposomes with the epithelial cells is anticipated to induce selectivity in the gene therapeutic treatment of the cancer cells. Figure 9B summarizes the cell viability results of the MCF-7 cancer cells *vs.* the MCF-10A epithelial breast cells treated with the x-modified liposomes loaded with the pre-irradiated hairpins (configuration I), entry (a) or with the x-modified liposomes loaded with the caged hairpin $H_{EE'}$ being intracellularly activated by light, (configuration II), entry (b). In these experiments, the cell viability of the respective cells were evaluated after fusion for a time-interval of four hours (For experimental details of the fusion process see experimental section, and Figure 9A) and allowing the operation of the DNAzyme cleaving EGR-1 mRNA process for a time-interval of 24 hours. Evidently, comparable results are observed by treatment of the cells using the two configurations of loaded liposomes. For the MCF-7 cells treated with the pre-irradiated liposomes, a ca. 75% cell death is observed, whereas the MCF-7 cells treated with intracellular light activation of the DNAzyme cleaving EGR-1 mRNA, a ca. 80% cell death is observed after this time-interval. For both systems, however, no noticeable cell death is observed for the epithelial MCF-10A cells, consistent with the lack of fusion of the liposomes

with these cells. Moreover, treatment of the MCF-7, MCF-10A cells with non-irradiated liposomes loaded with the caged hairpins $H_{EE'}$, non-modified with x, entry (c) or modified with x, entry (d), did not affect the cell viability, demonstrating that the light-induced unlocking of the hairpins is, indeed, essential to yield active DNAzyme cleaving EGR-1 mRNA. Furthermore, treatment of the cells with pre-irradiated caged hairpins $H_{EE'}$-loaded liposomes (configuration I) lacking tether x, entry (e), or treatment of cells loaded with caged hairpins $H_{EE'}$ (configuration II) lacking tether x, followed by irradiation of the cells, entries (e) and (f) show negligible affect on the cell viability, indicating that the x/x′-aptamer AS1411-induced fusion, indeed, activates the DNAzyme cleaving EGR-1 mRNA.

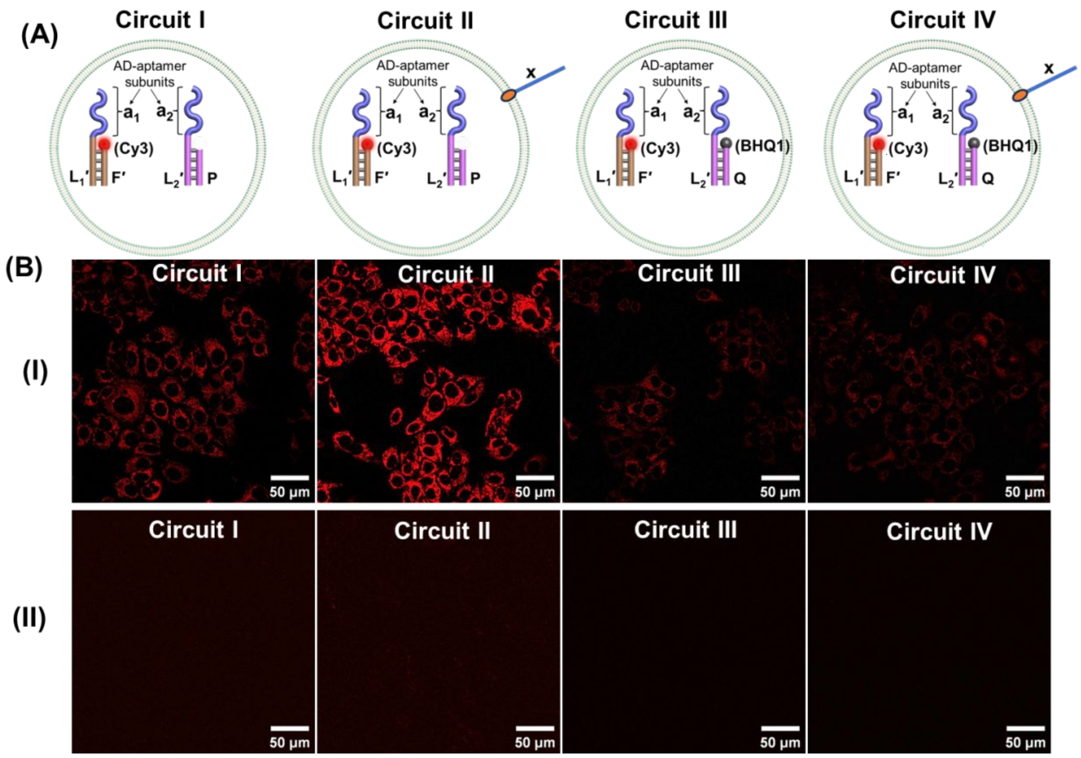

Figure S22. (A) Four different configurations of circuit-loaded lipsomes for probing the fusion effeciency between the module-loaded liposomes and cells (MCF-7 breast cancer cells and MCF-10A epithilial breast cells): circuit I: liposome loaded with duplexes $L_1'F'$ (strand F′ labeled with fluorophore Cy3) and $L_2'P$; circuit II: x-modified liposome loaded with duplexes $L_1'F'$ (Strand F′ modified with fluorophore Cy3) and $L_2'P$; circuit III: liposome loaded with duplexes $L_1'F'$ and $L_2'Q$ (Strand Q modified with quencher BHQ1); circuit IV: x-modified liposome loaded with duplexes $L_1'F'$ and $L_2'Q$. (B) Intracellular fluorescent images of MCF-7 breast cancer cells (Panel I) and MCF-10A breast normal cells (Panel II) upon subjecting respective circuits I-IV-loaded liposomes to cells.

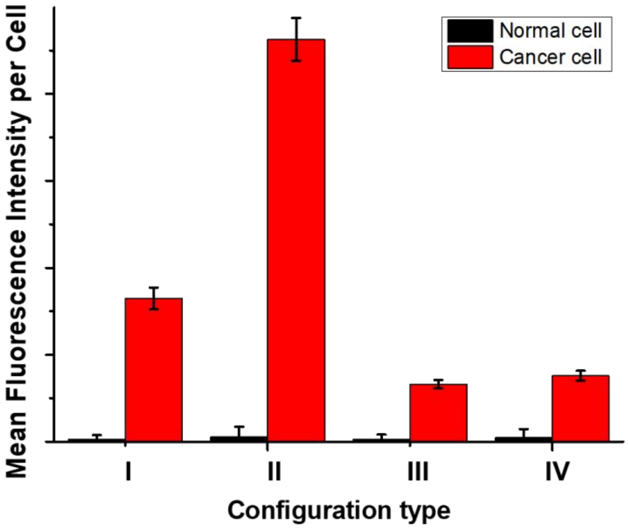

Figure S23. Summary intracellular fluorescence intensity analysis of MCF-7 breast cancer cells (red column) and MCF-10A breast normal cells (black column), shown in S22B, upon subjecting respective circuits I-IV-loaded liposomes to cells. Error bars derived from four experimental images.

Table S1. Rate constants derived from the computational simulation of AD-triggered transient reaction circuit shown in Figure 1.

k_1	29.1 μM^{-3} min^{-1}	k_2	201 μM^{-1} min^{-1}	k_5	92527 min^{-1}
k_{-1}	343.4 min^{-1}	k_{-2}	213 min^{-1}		

Table S2. Rate constants derived from the computational simulation of AD-triggered transient DNAzyme circuits shown in Figure 2.

k_1	21.3 μM^{-3} min^{-1}	k_2	201 μM^{-1} min^{-1}	k_5	92527 min^{-1}
k_{-1}	251.3 min^{-1}	k_{-2}	213 min^{-1}		

Table S3. Rate constants derived from the computational simulation of AD-triggered transient evolution of CDN X shown in Figure 3.

k_1	49.8 μM^{-2} min^{-1}	K_4	12.1 μM^{-2} min^{-1}	k_7	92527 min^{-1}
k_{-1}	587.6 min^{-1}	k_{-4}	142.8 min^{-1}		
k_2	29.3 μM^{-2} min^{-1}	k_5	3.6 μM^{-1} min^{-1}		
k_{-2}	345.7 min^{-1}	k_{-5}	7.2 μM^{-1} min^{-1}		
k_{-3}	11.2 μM^{-2} min^{-1}	k_{-6}	201 μM^{-1} min^{-1}		
k_{-3}	132.1 min^{-1}	k_6	213 min^{-1}		

Reference:

(1) Akbarzadeh, A.; Rezaei-Sadabady, R.; Davaran, S.; Joo, S. W.; Zarghami, N.; Hanifehpour, Y.; Samiei, M.; Kouhi, M.; Nejati-Koshki, K. Liposome: classification, preparation, and applications. *Nanoscale Res. Lett.* **2013,** *8*, 102.
(2) Parigoris, E.; Dunkelmann, D. L.; Murphy, A.; Wili, N.; Kaech, A.; Dumrese, C.; Jimenez-Rojo, N.; Silvan, U. Facile generation of giant unilamellar vesicles using polyacrylamide gels. *Sci. Rep.* **2020,** *10*, 4824.
